# Parasite Fate and Involvement of Infected Cells in the Induction of CD4^+^ and CD8^+^ T Cell Responses to *Toxoplasma gondii*

**DOI:** 10.1371/journal.ppat.1004047

**Published:** 2014-04-10

**Authors:** Christopher D. Dupont, David A. Christian, Elizabeth M. Selleck, Marion Pepper, Michael Leney-Greene, Gretchen Harms Pritchard, Anita A. Koshy, Sagie Wagage, Morgan A. Reuter, L. David Sibley, Michael R. Betts, Christopher A. Hunter

**Affiliations:** 1 Department of Pathobiology, School of Veterinary Medicine, University of Pennsylvania, Philadelphia, Pennsylvania, United States of America; 2 Department of Molecular Microbiology, Washington University School of Medicine, St. Louis, Missouri, United States of America; 3 Department of Immunology, University of Washington, Seattle, Washington, United States of America; 4 Department of Neurology, University of Arizona, Tucson, Arizona, United States of America; 5 Department of Microbiology, Perelman School of Medicine, University of Pennsylvania, Philadelphia, Pennsylvania, United States of America; University of Medicine and Dentistry of New Jersey, United States of America

## Abstract

During infection with the intracellular parasite *Toxoplasma gondii*, the presentation of parasite-derived antigens to CD4^+^ and CD8^+^ T cells is essential for long-term resistance to this pathogen. Fundamental questions remain regarding the roles of phagocytosis and active invasion in the events that lead to the processing and presentation of parasite antigens. To understand the most proximal events in this process, an attenuated non-replicating strain of *T. gondii* (the *cpsII* strain) was combined with a cytometry-based approach to distinguish active invasion from phagocytic uptake. In vivo studies revealed that *T. gondii* disproportionately infected dendritic cells and macrophages, and that infected dendritic cells and macrophages displayed an activated phenotype characterized by enhanced levels of CD86 compared to cells that had phagocytosed the parasite, thus suggesting a role for these cells in priming naïve T cells. Indeed, dendritic cells were required for optimal CD4^+^ and CD8^+^ T cell responses, and the phagocytosis of heat-killed or invasion-blocked parasites was not sufficient to induce T cell responses. Rather, the selective transfer of *cpsII*-infected dendritic cells or macrophages (but not those that had phagocytosed the parasite) to naïve mice potently induced CD4^+^ and CD8^+^ T cell responses, and conferred protection against challenge with virulent *T. gondii*. Collectively, these results point toward a critical role for actively infected host cells in initiating *T. gondii*-specific CD4^+^ and CD8^+^ T cell responses.

## Author Summary

CD4^+^ and CD8^+^ T cells are critical for controlling many infections. To generate a T cell response during infection, T cells must encounter the microbial peptides that they recognize bound to MHC molecules on the surfaces of other cells, such as dendritic cells. It is currently unclear how dendritic cells acquire the antigens they present to T cells during infection with many intracellular pathogens. It is possible that these antigens are phagocytosed and processed by dendritic cells, or antigens may be presented by cells that are infected by pathogens such as *Toxoplasma gondii*, which invades host cells independently of phagocytosis. To differentiate these pathways, we developed a novel technique to track the fate of *T. gondii* in vivo that distinguishes actively infected cells from those that phagocytosed parasites. This technique was used to examine each of these cell populations. We also used pharmacological inhibitors of parasite invasion, and the transfer of sort-purified infected or uninfected dendritic cells and macrophages to determine what roles phagocytosis and active invasion have in the initiation of T cell responses. Our results demonstrate that phagocytosis of parasites is not sufficient to induce CD4^+^ or CD8^+^ T cell responses, whereas infected cells are critical for this process.

## Introduction

*Toxoplasma gondii* is an intracellular protozoan parasite of medical and veterinary significance that can induce acute disease in its host and is an important opportunistic pathogen in immunocompromised individuals [1], [2]. Successful control of this pathogen requires a rapid T_H_1 immune response, characterized by the production of the cytokine IL-12, which promotes the ability of parasite-specific CD4^+^ and CD8^+^ T cells to produce the cytokine Interferon-γ (IFN-γ) [3], [4], [5]. The initiation of CD8^+^ T cell responses is a complex process which requires that professional antigen presenting cells acquire antigens and present them in the context of Major Histocompatibility Complex (MHC) I, and multiple models have been proposed to explain how this may occur during toxoplasmosis [6], [7]. For example, in other systems, foreign antigens are acquired through the pinocytosis of soluble antigens, the phagocytosis of large particulate antigens, or the phagocytosis of host cells containing foreign antigens, and subsequently presented to CD8^+^ T cells through cross-presentation [8], [9]. A role for cross presentation during toxoplasmosis is supported by in vivo imaging studies showing that uninfected dendritic cells interact extensively with parasite-specific CD8^+^ T cells [6], [10], [11]. Alternatively, since *T. gondii* is an intracellular parasite, actively infected dendritic cells may acquire parasite-derived antigens from their intracellular environment independently of phagocytosis and directly prime naïve CD8^+^ T cells. Indeed, the ability of cells actively infected by *T. gondii* to prime or present antigen to CD8^+^ T cells has been observed in vitro [12]–[14] and the critical role of perforin in immunity to *T. gondii* implicates the cytolysis of infected host cells as a mechanism of defense, thus arguing that infected cells can present antigen to effector CD8^+^ T cells in vivo [15]. However, several caveats must be acknowledged in interpreting these studies. Firstly, the ability of infected cells to present antigens to reporter cells lines or activated effector CD8^+^ T cells does not necessarily indicate that infected cells can prime naïve CD8^+^ T cells, and events that occur in vitro may not represent the in vivo situation. Additionally, it can be difficult to distinguish actively infected host cells from those that have phagocytosed the parasite by flow cytometry, thus confounding experimental interpretation. Furthermore, like many intracellular pathogens, *T. gondii* has been reported to inhibit the expression or upregulation of molecules involved in antigen presentation such as MHCI, CD40, CD80, and CD86 on infected cells, suggesting that the ability of infected cells to prime naïve CD8^+^ T cells may be compromised [16]–[18].

Antigens presented to CD4^+^ T cells in the context of MHCII may also be derived from the extracellular or intracellular environment of the host cell. Endocytosed antigens can be presented in the context of MHCII, and this pathway is considered to be the primary mechanism by which antigens are acquired for presentation to CD4^+^ T cells [19]. However, intracellular antigens can also be presented in the context of MHCII, as cytosolic peptides are presented in the context of MHCII by B cells and macrophages [20]. Similarly, in vitro studies have demonstrated that viral or model antigens expressed intracellularly can be presented to CD4^+^ T cells independently of phagocytosis [21]–[29]. Despite these findings, the role of infected cells in presenting antigen to CD4^+^ T cells in vivo during any infection remains unclear [30]. In the case of *T. gondii*, downregulated expression of MHCII and other molecules involved in antigen presentation has been observed on infected cells, and cells infected with *T. gondii* exhibit decreased ability to present antigen in vitro [16]–[18]. Furthermore, in vitro studies have observed that antigens from heat-killed or invasion-inhibited parasites incubated with dendritic cells can be presented in the context of MHCII, consistent with a role for phagocytosis-dependent antigen presentation to CD4^+^ T cells [12].

There are several difficulties involved with addressing the relative contributions of phagocytosis versus active invasion to antigen presentation in vivo during many infections. For example, interfering with these pathways can result in changes in pathogen burden and inflammation that confound experimental interpretation, and the parasite-mediated lysis of host cells and re-infection may obscure the analysis of the earliest cell populations that interact with the pathogen. In addition, there are limited tools to distinguish host cells that have phagocytosed pathogens from those that have been productively infected. In the present study, these issues are addressed using a non-replicating uracil auxotrophic vaccine strain of *T. gondii* (the *cpsII* strain) [31]–[33] and a novel assay that tracks the fate of parasites and distinguishes active invasion from phagocytosis in vivo. Using these approaches, *cpsII* parasites were found to infect large numbers of macrophages and dendritic cells, and dendritic cells were found to be necessary for optimal *cpsII*-induced CD4^+^ and CD8^+^ T cell responses. Infected dendritic cells displayed an activated phenotype, characterized by high levels of CD86 and MHCI expression, which was unique from the phenotype of dendritic cells that had phagocytosed *T. gondii*. Furthermore, the administration of heat-killed or invasion-blocked parasites did not induce CD4^+^ or CD8^+^ T cell responses, thus demonstrating that phagocytosis of parasites is insufficient to activate naïve T cells. Lastly, the selective transfer of infected dendritic cells or macrophages, but not those that had phagocytosed *T. gondii*, to naïve mice resulted in robust CD4^+^ and CD8^+^ T cell responses and protection from challenge with a virulent strain of *T. gondii*. These findings point toward a critical role for infected cells in initiating the adaptive immune response to *T. gondii*.

## Results

### Development of a system to distinguish phagocytosis of parasites from active invasion

**Figure 1:**
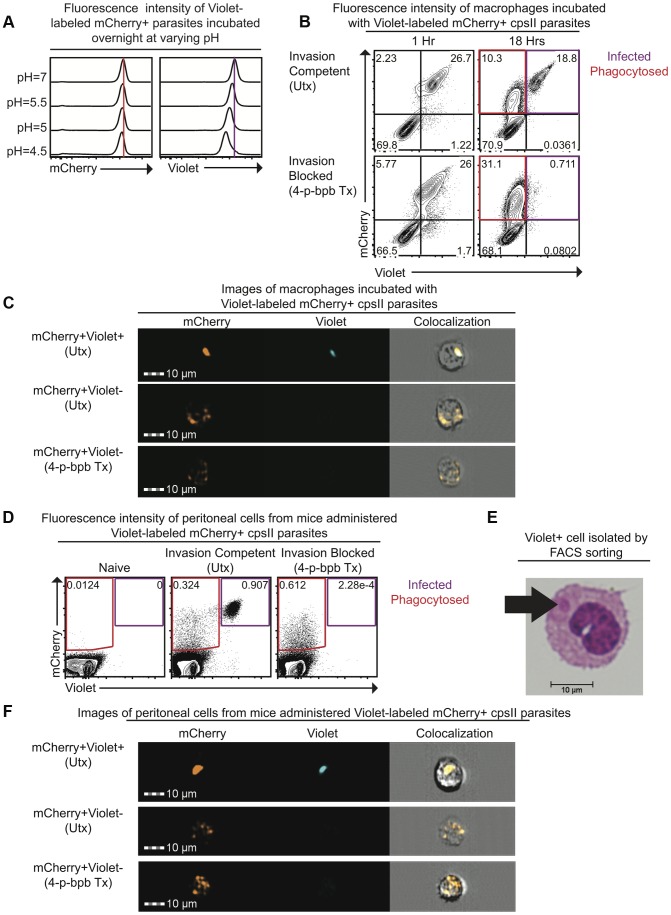
Differences in pH sensitivity of two fluorescent markers can be used to distinguish parasites that have been phagocytosed from those that actively invade host cells. Fluorescence intensity of mCherry-expressing *cpsII* parasites labeled with CellTrace Violet and incubated overnight at varying pH in buffer solutions consisting of citric acid and disodium phosphate [93] was measured by flow cytometry (a). Violet and mCherry fluorescence of immortalized murine bone marrow-derived macrophages exposed to Violet-labeled, mCherry-expressing *cpsII* parasites pre-treated with DMSO (top) or the irreversible inhibitor of invasion 4-p-bpb (bottom) 1 hour and 18 hours following exposure to parasites, measured by flow cytometry (b). Images of mCherry^+ve^Violet^+ve^ and mCherry^+ve^Violet^−ve^ bone marrow-derived macrophages 18 hours following exposure to Violet-labeled, mCherry-expressing *cpsII* parasites pre-treated with 4-p-bpb or DMSO (c). Violet and mCherry fluorescence of cells isolated from the PECS of mice 18 hours post-administration of 10^6^ DMSO-treated or 4-p-bpb-treated parasites (d). Cytospin analysis was performed on Violet^+ve^ cells isolated by FACS sorting, obtained from the PECS of a mouse 18 hours after vaccination with Violet-labeled *cpsII* parasites (e). Images of mCherry^+ve^Violet^+ve^ and mCherry^+ve^Violet^−ve^ cells isolated from the PECS of mice 18 hours post-administration of 10^6^ DMSO-treated or 4-p-bpb-treated Violet-labeled, mCherry-expressing *cpsII* parasites (f).

To distinguish between parasites that are phagocytosed by host cells and those that actively infect host cells, differences in sensitivity to pH between the fluorescent markers mCherry and CellTrace Violet were exploited. When mCherry-expressing parasites were labeled intracellularly with CellTrace Violet and incubated overnight in buffer solutions of varying pH, mCherry fluorescence was retained (Figure 1a). In contrast, violet fluorescence intensity was maintained at pH 7.0 but was decreased at low pH (Figure 1a). The ability of this system to distinguish active invasion from phagocytosis was demonstrated in vitro by incubating Violet-labeled, mCherry-expressing *cpsII* parasites with macrophages and examining fluorescence by flow cytometry 1 hour and 18 hours post-infection. At one hour after incubation with parasites, two distinct macrophage populations were present: One displayed mCherry and Violet fluorescence, while the other was negative for both markers (Figure 1b). However, by 18 hours, two distinct mCherry^+ve^ populations were apparent. One population displayed no loss of mCherry or Violet fluorescence (mCherry^+ve^Violet^+ve^), while the other population had decreased mCherry fluorescence associated with a complete loss of violet fluorescence (mCherry^+ve^Violet^−ve^). Utilizing ImageStream flow cytometry to generate images of individual cells from each of these populations revealed that the mCherry^+ve^Violet^+ve^ cells contained intact parasites, while the mCherry^+ve^Violet^−ve^ cells contained dimmer and more diffuse mCherry fluorescence (Figure 1c, Figure S1). Instances in which cells contained both diffuse fluorescence and intact parasites were rare (<3% of infected cells). Furthermore, pre-treatment of parasites with the irreversible inhibitor of invasion 4-p-bromophenacyl bromide (4-p-bpb) (thus making parasites targets for phagocytosis) [12], [34]–[36], resulted in the complete loss of the mCherry^+ve^Violet^+ve^ population at 18 hours post-infection (Figure 1b,c, Figure S1). Staining with LysoTracker, a fluorescent dye that specifically stains acidified compartments [37], enabled parasites that localized to acidified compartments to be distinguished from those that persist in non-acidified compartments. Both of these populations of parasites (LysoTracker^+ve^ and LysoTracker^−ve^) were apparent when untreated (invasion competent) parasites were incubated with bone marrow-derived macrophages one hour post-infection (Figure S2). In contrast, when invasion was pharmacologically inhibited parasites localized exclusively to the acidified compartments at these early time points, and at later time points the diffuse mCherry^+ve^ fluorescence localized most commonly to a LysoTracker^−ve^ compartment. Collectively, these results are consistent with a model in which phagocytosed parasites are degraded, and the acidic environment of the phagosome leads to a loss of Violet fluorescence, while mCherry fluorescence is retained. In contrast, when the parasite actively invades host cells and persists in the less acidic environment of the parasitophorous vacuole (PV), both Violet and mCherry fluorescence are retained.

The ability to distinguish active invasion from phagocytosis was then utilized to determine the fate of *cpsII* parasites in vivo. When C57BL/6 mice were vaccinated intraperitoneally with Violet-labeled, mCherry-expressing parasites, mCherry^+ve^Violet^+ve^ and mCherry^+ve^Violet^−ve^ populations were apparent in the Peritoneal Exudate Cells (PECS) 18 hours post-vaccination, and the presence of the mCherry^+ve^Violet^+ve^ population was abrogated by pre-treating the parasites with 4-p-bpb (Figure 1d). Furthermore, when Violet^+ve^ cells were sorted and cytospins were examined, they were found to contain intact parasites (Figure 1e). ImageStream analysis also revealed that the mCherry^+ve^Violet^+ve^ population contained intact parasites whereas the mCherry^+ve^Violet^−ve^ population displayed diffuse mCherry fluorescence (Figure 1f, Figure S3). Collectively, these studies demonstrate that the use of fluorescent markers with differing pH sensitivities can be used to distinguish cells that have phagocytosed *T. gondii* from those that have been actively infected.

### *CpsII* parasites can persist within infected host cells, but are ultimately cleared from the peritoneal cavity

To measure the persistence of *cpsII* parasites in vivo, bioassays were performed in which tissues from vaccinated mice were cultured in the presence of exogenous uracil and examined by microscopy for the presence of *cpsII* parasites. Using this method, *cpsII* parasites were detected in all mice examined at day 3 post-infection. However, by day 5 post-infection, 50% of mice had cleared the infection, and by day 10 post-infection, no parasites could be detected. These data suggest that *cpsII* parasites are ultimately cleared from the host, and are consistent with previous studies, in which parasite DNA could not be detected in the peritoneal cavities or spleens of *cpsII*-vaccinated mice when measured 3 weeks post-infection [38].

**Figure 2:**
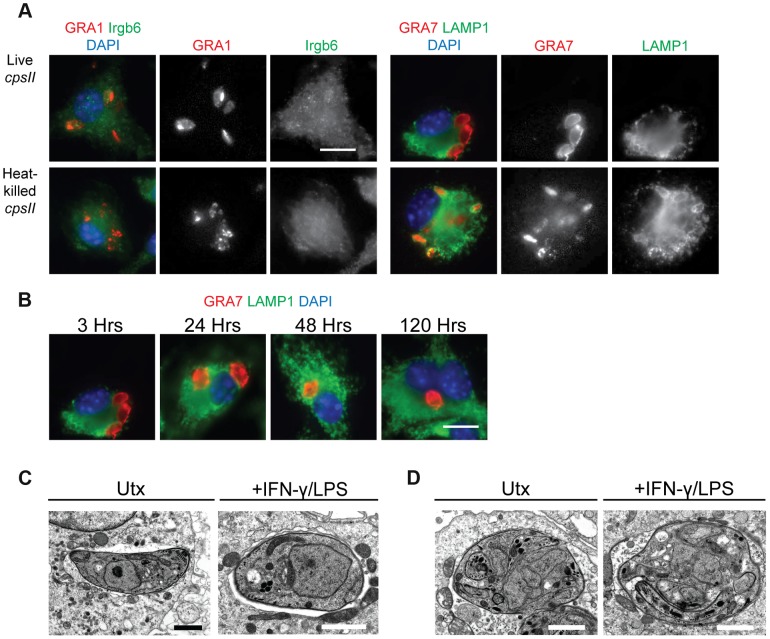
The fate of heat-killed and live *cpsII* parasites in host cells. C57BL/6 bone marrow derived macrophages were infected with *cpsII* parasites and examined using immunofluorescence assays (a). Bone marrow-derived macrophages activated with IFN-γ (100 U/ml) and LPS (0.1 ng/ml) were infected with freshly lysed or heat-killed parasites for 3 hours. Intracellular parasites were stained for host Irgb6 or LAMP1 recruitment in green. Parasites were stained with a mouse monoclonal antibody to GRA1 to identify the parasitophorous vacuole or rabbit polyclonal sera against GRA7 in red. IFN-γ and LPS activated bone marrow-derived macrophages were infected with freshly lysed *cpsII* parasites and fixed at 3, 24, 48 and 120 hours post-infection (b). Parasite vacuoles were identified with rabbit polyclonal sera to GRA7 (red) and host LAMP1 was identified with a rat monoclonal antibody. Scale bar = 10 µm. Electron micrograph images of infected macrophages treated with IFN-γ (50 units/ml) and LPS (10 ng/ml) or untreated at 2 hours post-infection (c). Parasites persist in intact vacuoles and do not display blebbing or disruption of the parasitophorous vacuole. Some *cpsII* parasites were found to exhibit non-productive cell division in IFN-γ and LPS- treated or untreated macrophages when examined 24 hours post-infection (d). Scale bars = 1.5 µm.

To determine the mechanisms by which *cpsII* parasites may ultimately be cleared from host cells, their fate within infected host cells was examined in vitro. Since IFN-γ (in combination with LPS or TNF-α) can induce the recruitment of immune enzymes such as the Immunity Related Guanosine Triphosphatases (IRGs) to the PV, and these enzymes have been implicated in the rupture of the PV which leads to the xenophagic elimination of the parasite [39], the colocalization of the parasite with Irgb6 (a member of the IRG family) and LAMP-1 (which is expressed on lysosomes) in IFN-γ–activated cells and untreated cells was examined using immunofluorescence microscopy, to determine if IFN-γ induced the elimination of *cpsII* parasites within infected cells. When the subcellular localization of live *cpsII* parasites was examined, it was apparent that these parasites did not colocalize with either Irgb6 or LAMP-1 in IFN-γ-activated or untreated macrophages, at any time point examined (ranging from 3 hours post-infection to 5 days post-infection) (Figure 2a–b). In contrast, LAMP-1 colocalized with heat-killed parasites, consistent with the idea that heat-killed parasites are phagocytosed. These data argue against the notion that *cpsII* parasites are eliminated by xenophagy, and demonstrate that these parasites can persist within infected cells for long periods of time. Electron microscopy was also utilized to examine the integrity of the PV, since IFN-γ can induce the blebbing and rupture of the PV during infection with replicating strains of *T. gondii* [40], [41]. Using this approach, *cpsII*-infected macrophages were consistently observed to contain intact PVs and blebbing was not apparent (Figure 2c). Additionally, some *cpsII* parasites showed atypical morphology, indicative of non-productive cell division (Figure 2d). Collectively, these results confirm that *cpsII* parasites cannot replicate within host cells, and suggest that *cpsII* parasites can persist within infected cells, evading IFN-γ-mediated destruction, although they are eventually cleared from the host.

### Identification and phenotypic analysis of cells that are infected by or phagocytose *cpsII* parasites

**Figure 3:**
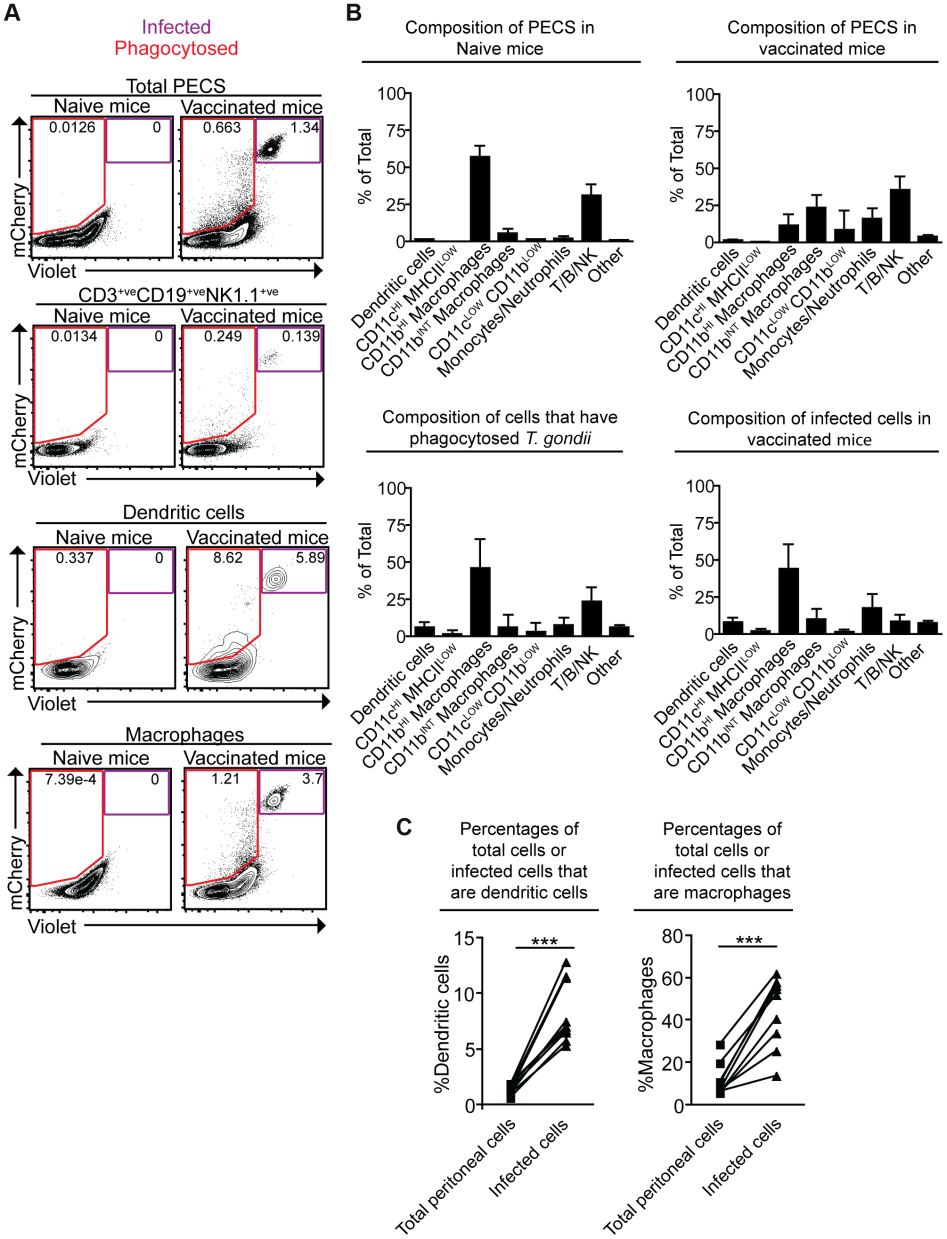
Composition of total cell populations, mCherry^+ve^Violet^−ve^ cell populations, and mCherry^+ve^Violet^+ve^ populations from the PECS of naïve and vaccinated mice. Mice were vaccinated with 10^6^ Violet-labeled, mCherry-expressing *cpsII* parasites intraperitoneally and sacrificed 18 hours post-vaccination. Cell type composition of total peritoneal cell populations in naïve and vaccinated mice, and the cell type composition of mCherry^+ve^Violet^−ve^ cells and mCherry^+ve^Violet^+ve^ cells in vaccinated mice were examined. Representative flow plots demonstrating infected cells and cells that have phagocytosed *T. gondii* for each major cell type present in the PECS are shown (a). The composition of the PECS in naïve mice and vaccinated mice, and the composition of infected cells (mCherry^+ve^Violet^+ve^) and cells that have phagocytosed *T. gondii* (mCherry^+ve^Violet^−ve^) are depicted (b). Percentages of macrophages and dendritic cells in the total peritoneal cell population in vaccinated mice are compared to the percentages of infected cells that are macrophages and dendritic cells (c). T/B/NK cells are identified by expression of CD3, CD19, or NK1.1. Dendritic cells were identified as CD3^−ve^,CD19^−ve^,NK1.1^−ve^,CD11c^HI^,MHCII^HI^. Monocytes and neutrophils were defined as CD3^−ve^,CD19^−ve^,NK1.1^−ve^,CD11c^LOW-INT^,Gr-1^+ve^. Macrophages were identified as CD3^−ve^,CD19^−ve^,NK1.1^−ve^,CD11c^LOW-INT^,Gr-1^−ve^,CD11b^INTorHI^. *p<0.05; ***p<0.0005. AVG±STDEV. A paired, two-tailed student's t test was used to analyze the data in (c). Results shown are from one representative experiment. Similar results were obtained over the course of seven separate experiments.

To better understand the fate of *cpsII* parasites in vivo, mice were challenged intraperitoneally with Violet-labeled, mCherry-expressing *cpsII* parasites, and flow cytometry was performed on the PECS 18 hours later to characterize the cell populations that had phagocytosed *T. gondii* or were actively infected. The largest population of mCherry^+ve^Violet^+ve^ cells to be infected was CD11b^HI^ macrophages, which comprised 44.0±16.7% of infected cells. Dendritic cells (which have been previously implicated in the induction of T cell responses to *cpsII* [42]) comprised 8.3±2.8% of infected cells (Figure 3a,b). Of the infected dendritic cells the vast majority (97.8±2.0%) belonged to the Gr-1^−ve^CD11b^HI^ subset (data not shown). Although *T. gondii* is capable of infecting any nucleated cell, when the frequencies of CD11b^HI^ macrophages and dendritic cells within the population of infected cells (44.0±16.7% and 8.3±2.8%, respectively) were compared to their frequencies within the total population of peritoneal cells in vaccinated mice (11.3±7.9% and 1.3±0.4%, respectively), it was apparent that macrophages and dendritic cells are overrepresented among cells infected by the parasite (Figure 3c). Analysis of the population that had phagocytosed *T. gondii* revealed 46.0±20.6% of these cells were CD11b^HI^ macrophages, whereas dendritic cells represented 6.2±3.2% of this population (Figure 3a,b). Additionally, 23.4±9.9% of the cells that had phagocytosed the parasite stained positive for markers for T, B or NK cells (CD3, CD19 and NK1.1, respectively). Further sub-setting revealed these cells to be B cells, consistent with previous reports identifying a population of phagocytic B cells in the peritoneal cavity (Figure 3b, data not shown) [43], [44]. Parasites were not detected in lymph nodes or spleens by flow cytometry, and parasites could not be cultured from these tissues at days 3,5 or 10 post-vaccination.

**Figure 4:**
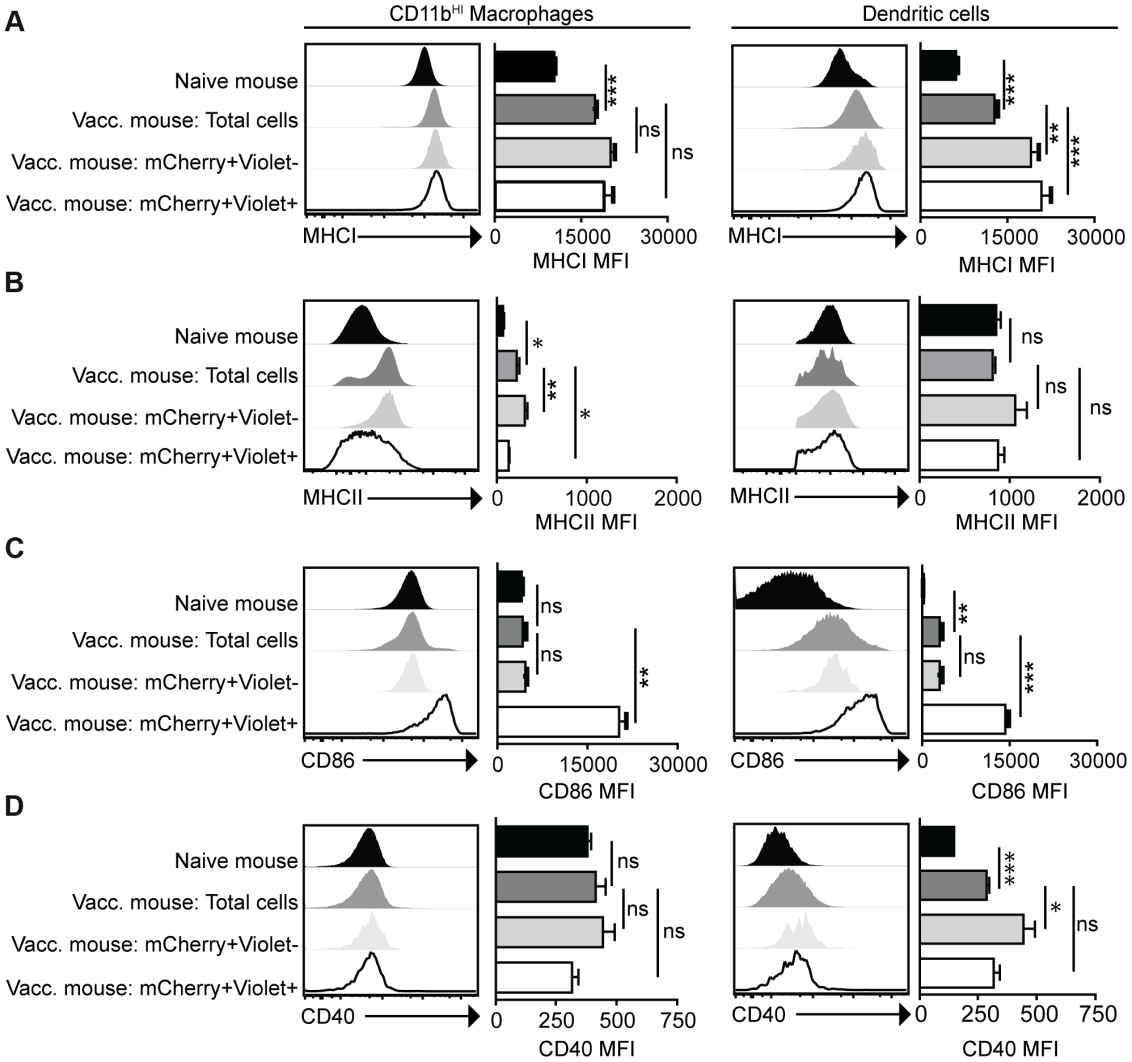
Activation status of mCherry^+ve^Violet^−ve^ and mCherry^+ve^Violet^+ve^ macrophages and dendritic cells. Mice were administered parasites as described in Figure 3. At 18 hours post-vaccination, expression of the antigen presentation molecules MHCI (a) and MHCII (b) and expression of the costimulatory molecules CD86 (c) and CD40 (d) on CD11b^HI^ macrophages and dendritic cells was determined by flow cytometry. Macrophages are identified as CD3^−ve^,CD19^−ve^,NK1.1^−ve^,CD11c^−ve^,Gr-1^−ve^,CD11b^HI^ cells. Dendritic cells are identified as CD3^−ve^,CD19^−ve^,NK1.1^−ve^,CD11c^HI^,MCHII^HI^. Confidence intervals were determined using the Bonferroni correction method. *p<0.017; **p<0.0017; ***p<0.00017. AVG±SE. Paired, two-tailed student's t tests were used to compare expression levels of molecules on populations within *cpsII*-vaccinated mice.

The phenotype of infected cells and those that phagocytosed the parasite was compared by analyzing expression levels of MHCI and MHCII, as well as the costimulatory molecules CD86 and CD40. Although vaccination with *cpsII* resulted in an overall increase in expression of MHCI on CD11b^HI^ macrophages, macrophages that had phagocytosed the parasite and those that were infected displayed similar levels of MHCI to the total population present in the PECS of vaccinated mice. In contrast, dendritic cells that had phagocytosed *cpsII* and those that were infected by the parasite displayed higher levels of MHCI relative to the total dendritic cell population in the peritoneal cavity (Figure 4a). Vaccination with *cpsII* induced no significant changes in MHCII expression on dendritic cells, although infected macrophages had lower levels of MHCII than the total population in the PECS (Figure 4b). Expression of CD86 was markedly higher on macrophages and dendritic cell populations that were infected by the parasite, but not the populations that had phagocytosed the parasite (Figure 4c). While vaccination induced increased CD40 expression on the total dendritic cell population, infected cells displayed similar expression levels to the total population, and those that phagocytosed the parasite exhibited the highest levels of expression (Figure 4d). Collectively, these results reveal a complex pattern demonstrating that infected macrophages and dendritic cells display activated phenotypes, characterized by the upregulation of MHCI and CD86, and constitutive expression of CD40 and MHCII, which is distinct from the phenotype of cells that phagocytosed *T. gondii*.

### Dendritic cells are critical for optimal *cpsII*-induced CD4^+^ and CD8^+^ T cell responses

**Figure 5:**
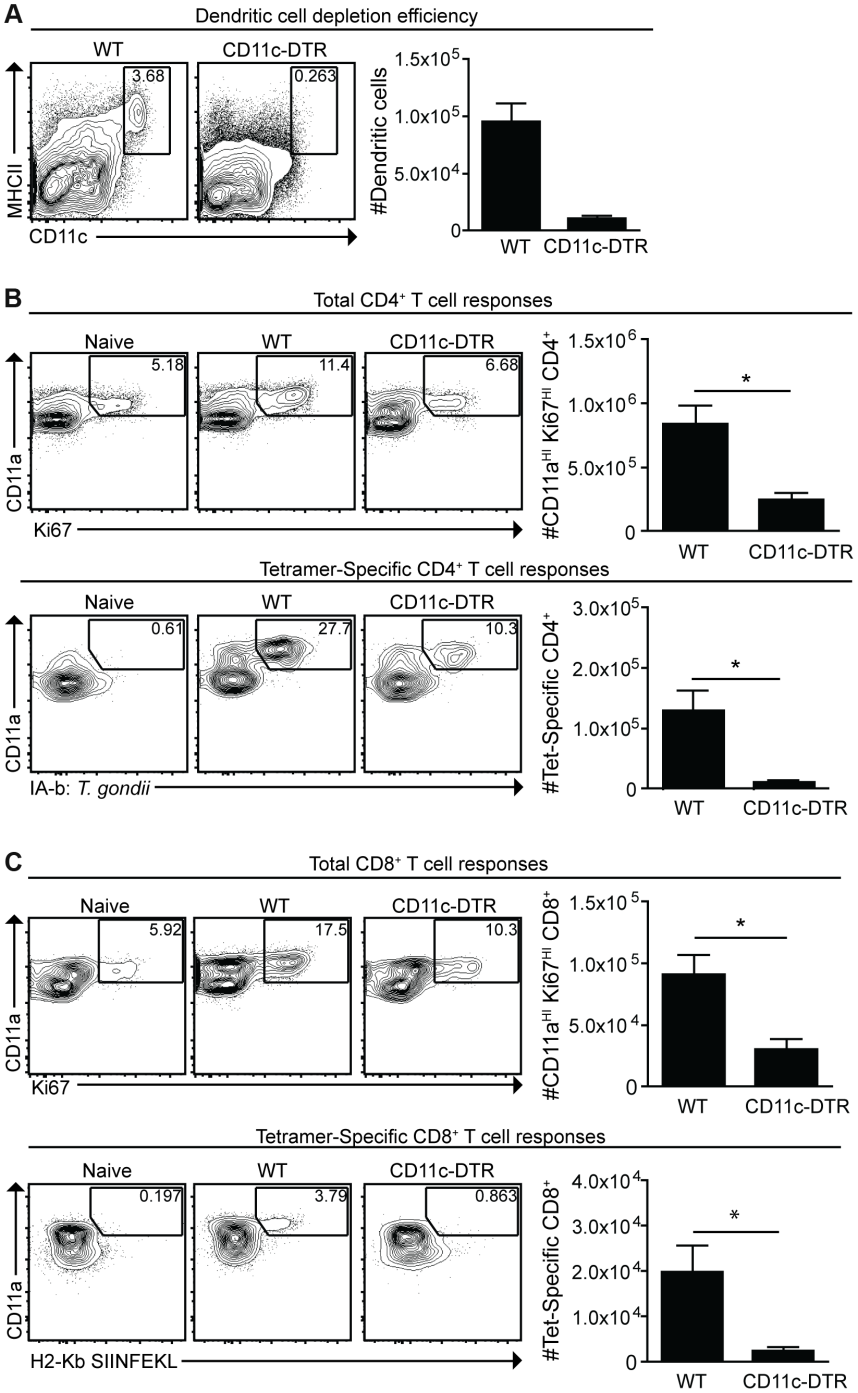
Dendritic cells are required for optimal CD4^+^ and CD8^+^ T cell responses. CD11c-DTR mice were administered diphtheria toxin 1 day prior to *cpsII*-OVA vaccination. At the time of vaccination, some mice were sacrificed to determine the efficiency of depletion. Percentages and numbers of dendritic cells from the spleen are shown. FACS plots are gated on CD3^−^,CD19^−^,NK1.1^−^ cells (a). Eight days following vaccination, mice were sacrificed and total and tetramer-specific CD4^+^ and CD8^+^ T cell responses were analyzed. Total CD4^+^ T cell responses from the spleens are shown (b, top). Tetramer-specific CD4^+^ T cell responses from pooled lymph nodes and splenocytes were determined in a separate experiment (b, bottom). Flow plots are gated on CD4^+^ T cells (b), and the population examined was magnetically enriched for the tetramer^+ve^ population (b, bottom). Total and OVA-specific CD8^+^ T cell responses from the PECS are depicted (c), and flow plots are gated on CD8+ T cells. Significant differences in tetramer and total CD8^+^ T cell responses between WT and CD11c-DTR mice were also apparent in the spleen. *p<0.05; **p<0.005. AVG±SE.

Given the activated phenotype of dendritic cells infected with *cpsII* versus those that had phagocytosed the parasite, studies were performed to determine the role of dendritic cells in the development of CD4^+^ and CD8^+^ T cell responses to this strain. Mice that express the diphtheria toxin receptor under the control of the CD11c promoter (CD11c-DTR mice) were used to test the requirement for dendritic cells to prime T cells [45]. In these experiments, CD11c-DTR mice were treated with diphtheria toxin, which resulted in a 70–90% reduction in dendritic cells (Figure 5a). One day following the administration of diphtheria toxin, mice were challenged with a strain of *cpsII* engineered to express Ovalbumin (*cpsII*-OVA) [38]. At eight days following vaccination, CD4^+^ and CD8^+^ T cell responses were measured using MHCII tetramers, which bind CD4^+^ T cells specific for the endogenous *T. gondii* epitope CD4Ag28m combined with magnetic enrichment for the tetramer^+ve^ population [46], [47], and MHCI tetramers for OVA-specific CD8^+^ T cells. Additionally, the surface molecule CD11a, which is upregulated on antigen-experienced CD4^+^ and CD8^+^ T cells [48], [49], and the intracellular molecule Ki67, which is indicative of cellular proliferation [50], were used to estimate the total CD4^+^ and CD8^+^ T cell responses to *T. gondii*. Indeed, vaccination with *cpsII* induced a two-fold increase in the frequency of CD11a^HI^Ki67^HI^ cells and an expansion in the number of CD11a^HI^ CD4^+^ T cells specific for the CD4Ag28m epitope, but depletion of dendritic cells inhibited these responses (Figure 5b). Similarly, *cpsII* vaccination induced an increase in CD11a^HI^Ki67^HI^ and OVA-specific CD8^+^ T cells, however these responses were decreased in mice depleted of dendritic cells (Figure 5c). Furthermore, when Flt3L^−/−^ mice (which have global defects in numbers of dendritic cells [51]) or Batf3^−/−^ mice (which have a defect in numbers of CD8a^+^ dendritic cells [52]) were challenged with *cpsII*-OVA, both mice displayed marked defects in tetramer-specific and total CD4^+^ and CD8^+^ T cell responses (Figure S4,S5).

Given the numbers of macrophages that were either infected or which had phagocytosed *T. gondii*, experiments were performed to assess their role in the *cpsII*-induced T cell responses. However, attempts to deplete macrophages using clodronate liposomes also resulted in significant depletion of dendritic cells, making it difficult to assess the specific contribution of macrophages (data not shown). However, because monocytes were observed to interact with parasites (Figure 3b), and these populations can develop into dendritic cells that express CD11c, experiments were performed to assess their role in generating CD4^+^ and CD8^+^ T cell responses following *cpsII* vaccination. Therefore, mice deficient in the chemokine receptor CCR2, which promotes the recruitment of inflammatory monocytes to sites of inflammation during toxoplasmosis [53], were immunized with *cpsII*-OVA parasites. Despite having a defect in monocyte recruitment to the peritoneum, CCR2^−/−^ mice had similar *cpsII*-induced CD4^+^ and CD8^+^ T cell responses to WT control mice (Figure S6), thus arguing against a critical role for inflammatory monocytes in presenting antigen to CD4^+^ and CD8^+^ T cells following *cpsII*-vaccination. Collectively, these results establish a role for dendritic cells in the generation of CD4^+^ and CD8^+^ T cell responses following *cpsII* vaccination.

### Infected dendritic cells are sufficient to generate CD4^+^ and CD8^+^ T cell responses

**Figure 6:**
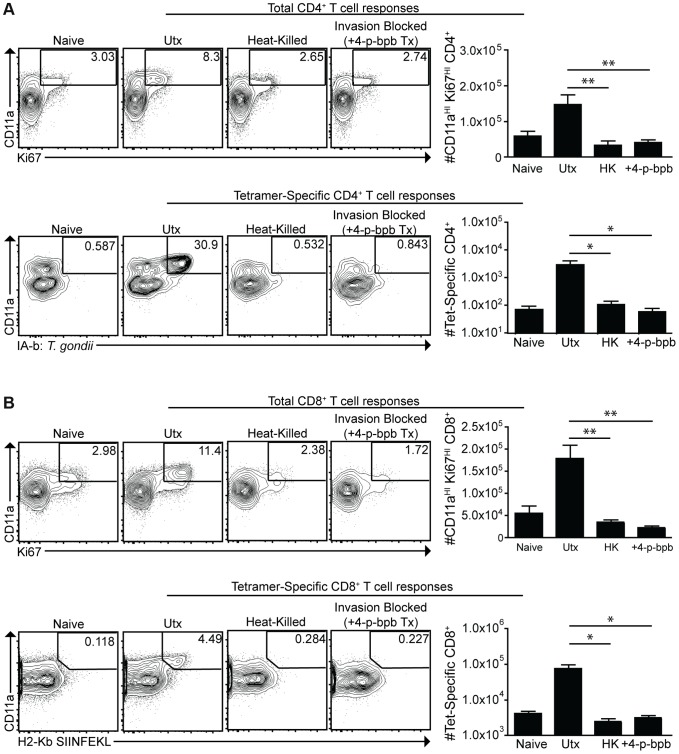
Active invasion is required for adaptive immune responses to *T. gondii*. *cpsII*-OVA parasites were heat-killed, treated with the invasion inhibitor 4-p-bpb or left untreated and administered to mice intraperitoneally. Tetramer-specific and total CD4^+^ (a) and CD8^+^ (b) T cell responses were measured from cells isolated from the spleen and lymph nodes (pooled) 10 days post-vaccination. Flow plots are gated on Foxp3^−ve^ CD4^+^ T cells (a, top) or CD4^+^ T cells (a, bottom) and the population examined at the bottom of A was enriched for tetramer^+ve^ cells. Flow plots in B are gated on CD8^+^ T cells. *p<0.05; **p<0.005. AVG±SE.

To assess the contribution of phagocytosis to the generation of CD4^+^ and CD8^+^ T cell responses, mice were challenged with live *cpsII*-OVA parasites, heat-killed *cpsII*-OVA parasites, or parasites pre-treated with the irreversible inhibitor of invasion 4-p-bpb. As expected, vaccination with live parasites induced a robust CD4^+^ T cell response, however these responses were abrogated when parasites were killed or invasion was inhibited (Figure 6a). Similarly, CD11a^HI^Ki67^HI^ and OVA-specific CD8^+^ T cells were detected when mice were administered live, but not heat-killed or invasion-inhibited parasites (Figure 6b). Indeed, even when the dose of heat-killed parasites was increased to 10^7^ parasites (100× the typical dose of live parasites used in these experiments), no CD4^+^ or CD8^+^ T cell responses could be detected (Figure S7). Additionally, gp91^−/−^ mice, which have a defect in cross-presenting antigens to CD8^+^ T cells [54], developed normal CD8^+^ T cell responses following *cpsII*-vaccination (data not shown). Collectively, these data indicate that phagocytosis of parasites is insufficient to induce CD4^+^ and CD8^+^ T cell responses, and point toward a critical role for infected cells in these processes.

**Figure 7:**
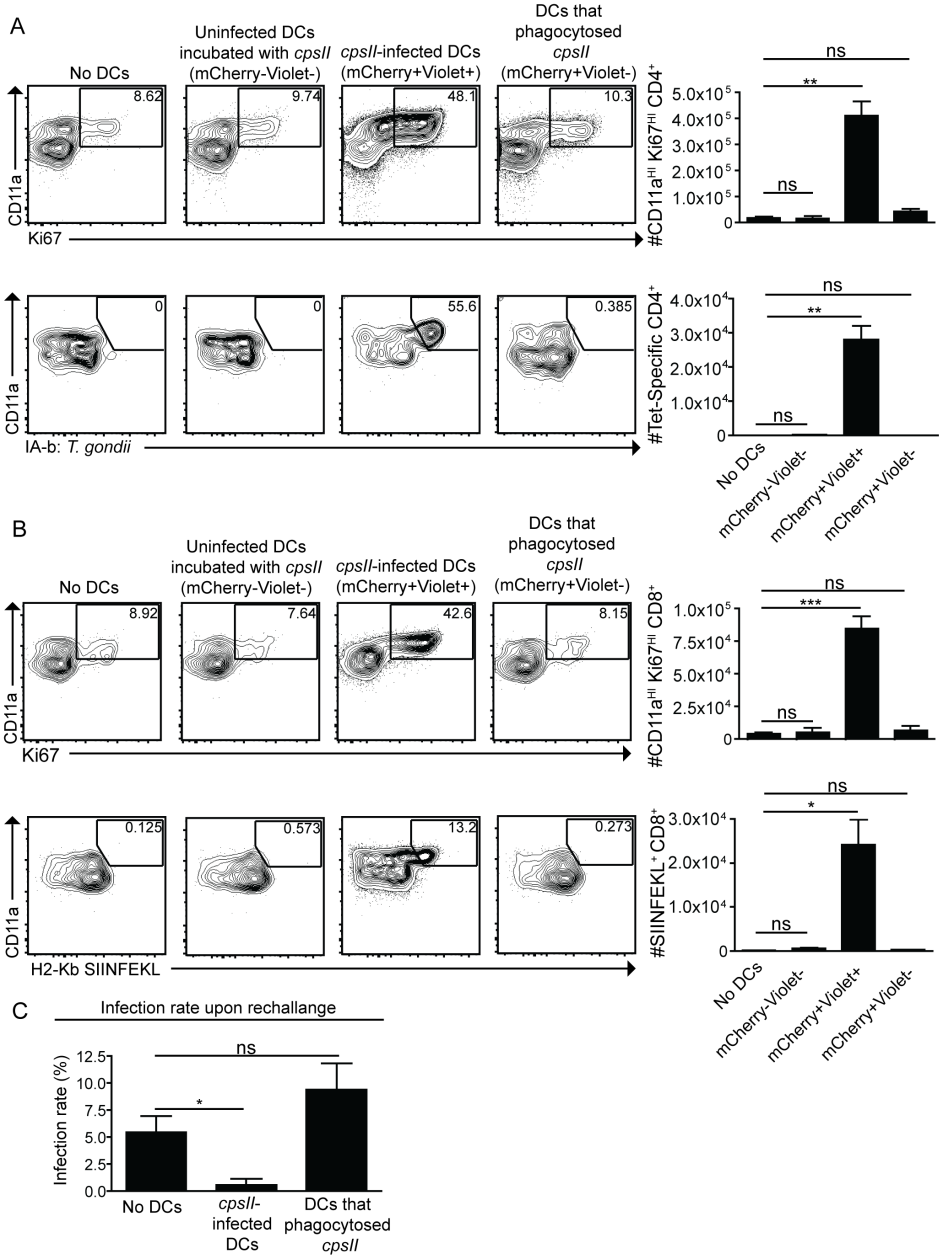
Infected cells are sufficient to induce CD4^+^ and CD8^+^ T cell responses. Bone marrow-derived dendritic cells were cultured overnight with Violet-labeled, mCherry-expressing *cpsII* parasites. The following day, dendritic cells were sorted into mCherry^+ve^Violet^+ve^ (infected) and mCherry^+ve^Violet^−ve^ (uninfected) populations and 10^4^ dendritic cells from each population were administered to mice. In parallel, dendritic cells that had phagocytosed *T. gondii* were obtained by sorting on bone marrow-derived dendritic cells that were incubated with invasion-blocked Violet-labeled, mCherry-expressing *cpsII* parasites. 10 days later, mice were sacrificed and CD4^+^ (a) and CD8^+^ (b) T cell responses in the peritoneal cavity and spleen were analyzed. Populations shown depicting CD4^+^-tetramer binding are enriched for the tetramer^+ve^ population and these cells were isolated from the spleen (a, bottom). All other cell populations shown were harvested from the peritoneal cavity, although similar trends were apparent when splenocytes were examined. Flow plots are gated on Foxp3^−ve^ CD4^+^ T cells (a, top), CD4^+^ T cells (a, bottom) or CD8^+^ T cells (b). Parasite burdens from the PECS of mice transferred infected dendritic cells or dendritic cells that have phagocytosed *T. gondii* 5 days post-challenge with 10^3^ tachyzoites of a highly virulent, replicating strain of *T. gondii*, administered 6 weeks following vaccination with 10^4^ infected or uninfected dendritic cells, analyzed by flow cytometry (c). Significance in (c) was determined using a Mann-Whitney U-test. *p<0.05; **p<0.005. AVG±SE.

To determine whether infected dendritic cells were sufficient to generate CD4^+^ and CD8^+^ T cell responses, bone marrow-derived dendritic cells cultured in GM-CSF (which are CD11b^HI^CD8α^−ve^) were infected with violet-labeled, mCherry-expressing *cpsII* parasites in vitro overnight, and FACS sorting was used to purify the uninfected (mCherry^−ve^Violet^−ve^) and infected cells (mCherry^+ve^Violet^+ve^) from the same cultures, and each of these fractions was then administered to naïve mice. In addition, bone marrow-derived dendritic cells were cultured with invasion-blocked parasites, and the populations of DCs that had phagocytosed the parasite (mCherry^+ve^Violet^−ve^) were also isolated by FACS sorting, and administered to mice. This experiment allowed a direct comparison of the ability of infected dendritic cells and dendritic cells that phagocytosed *T. gondii* to induce CD4^+^ and CD8^+^ T cell responses in vivo. In mice administered uninfected dendritic cells cultured with parasites, or dendritic cells that had phagocytosed parasites, there was no detectable increase in Ki67^+ve^CD11a^HI^, antigen-experienced CD4^+^ or CD8^+^ T cells (Figure 7a,b). In contrast, mice administered *cpsII*-infected dendritic cells developed CD4^+^ and CD8^+^ T cell responses as determined by tetramer-binding as well as expression of Ki67 and CD11a (Figure 7a,b). Furthermore, when vaccinated mice were challenged 6 weeks later with a highly virulent strain of *T. gondii*, only those mice administered *cpsII*-infected dendritic cells displayed a ∼90% reduction in parasite burden (Figure 7c). Similar results were obtained using splenic dendritic cells, which are composed of both CD8α^+^ and CD8α^−^ dendritic cells (data not shown). Moreover, the transfer of sort-purified infected bone marrow-derived macrophages to mice also induced CD4^+^ and CD8^+^ T cell responses and protected mice from challenge, whereas the transfer of macrophages that had phagocytosed parasites did not induce T cell responses or protection (Figure S8). Collectively, these results demonstrate a key role for infected cells in the induction of CD4^+^ and CD8^+^ T cell responses, and protective immunity upon re-challenge.

## Discussion

There are many fundamental questions about the mechanisms of antigen presentation that lead to the activation of CD4^+^ and CD8^+^ T cells during toxoplasmosis and multiple studies have addressed the ability of actively infected cells to present antigen [12]–[14], [55]. The present work highlights that following challenge in vitro or in vivo with live parasites there are high rates of phagocytosis and the combination of flow cytometry and parasites that express a single fluorescent reporter protein are not sufficient to distinguish infected cells from those that phagocytose *T. gondii*. Rather, the ability to combine parasites that express a pH insensitive reporter such as mCherry protein with a pH sensitive dye and analysis by high throughput imaging and flow cytometry provide a unique opportunity to examine parasite fate and host cell phenotype. This approach should be broadly applicable to determining the fate of other intracellular fungal, bacterial and parasitic pathogens [56]–[62]. Regardless, the ability to distinguish active invasion from phagocytosis revealed that macrophages and dendritic cells infected by *T. gondii* have unique activation phenotypes when compared to those that have phagocytosed the parasite. Previous reports have indicated that infection with *T. gondii* inhibits the maturation of professional antigen presenting cells [6], [16], [18], [63], but the data presented here are more consistent with the idea that infection induces DC maturation [36], [55], [64]–[66]. The experiments in which dendritic cells were selectively depleted, or pre-infected dendritic cells were transferred to mice highlight the important role of these accessory cells in generating CD4^+^ and CD8^+^ T cell responses following *cpsII*-vaccination. However, these findings do not rule out the possibility that other cell types are also involved. Indeed, the transfer of infected bone marrow-derived macrophages could also induce CD4^+^ and CD8^+^ T cell responses, suggesting that resident macrophages may also contribute to the T cell responses that occur following *cpsII* vaccination.

In current paradigms, the direct phagocytosis or endocytosis of soluble and particulate non-infectious antigens is the major pathway that allows antigens to be presented in the context of MHCII to CD4^+^ T cells [19]. Similarly, phagocytosed antigens are thought to be presented to CD8^+^ T cells through the process of cross-presentation [8]. However, the multiple approaches presented here indicate that phagocytosis of *T. gondii* is not sufficient to generate T cell responses. The finding that infected dendritic cells and macrophages display activated phenotypes and are able to promote CD4^+^ and CD8^+^ T cells responses in vivo distinguishes them from populations that phagocytose *T. gondii*. These observations suggest that live (as opposed to phagocytosed) parasites may uniquely activate innate sensing mechanisms that are linked to antigen presentation. This may relate to the persistence of parasites that occurs in infected cells, or to the engagement of mechanisms that allow the host to distinguish viable parasites from those that had been phagocytosed and would be killed [67]. The failure of cells that phagocytose the parasite to upregulate expression of CD86 is consistent with this idea. Another possibility is that dendritic cells actively infected with *T. gondii* display a hypermotile phenotype and enhanced migration to lymph nodes, a process that is considered essential for T cell priming [68]–[72]. Differences in cellular motility between infected cells and those that phagocytose parasites may account for the apparent discrepancy between the previous studies that showed that phagocytosis of parasites is sufficient to prime CD4^+^ T cells in vitro [12] and our finding that this process is not sufficient in vivo.

Regardless of the reasons that cells that phagocytose *T. gondii* fail to prime T cells, the data presented here are consistent with models in which infected cells either directly prime CD4^+^ T and CD8^+^ T cells, or are taken up by efferocytosis (i.e. the phagocytosis of apoptotic cells), leading to antigen presentation. Since *T. gondii* resides in a specialized non-fusogenic vacuole, it is unclear how parasite antigens may escape the PV for processing and presentation by infected cells. One possibility is that parasite antigens are acquired for presentation from the intracellular environment through the xenophagic elimination of *cpsII* parasites. Indeed, autophagic machinery has been implicated in the elimination of *T. gondii* [40], [73], [74], and antigen acquired through autophagy can be subsequently presented [23], [24], [75]. However, the lack of recruitment of Irgb6 and LAMP-1 to the PVs containing *cpsII* parasites argues against this idea. Other possible mechanisms that would allow parasite material to enter antigen processing pathways include the fusion of the PV with the endoplasmic reticulum [12], the secretion of antigen into the cytoplasm during invasion [76], or leakage of antigen out of the PV [14]. More recent work has shown that *T. gondii* can secrete antigens into host cells without subsequently infecting these cells [77]. This population of injected-but-uninfected cells may also contribute to the host immune response, and the ability to track these abortive invasion events in vivo, as well as the ability to divorce injection from infection through modulation of the parasite, may provide further insight into the pathways involved in antigen processing during *cpsII* vaccination.

Given the lack of overt inflammation observed during infection with *cpsII* parasites, the absence of parasite-driven cytolysis of host cells, and limited antigen load, it remains surprising that relatively low numbers of these parasites are able to generate strong protective CD4^+^ and CD8^+^ T cell responses, comparable to those seen during live infection [31]–[33], [38], [42], [78]. Increased antigenic burden is generally associated with increased T cell responses, and inflammatory signals can promote pathways involved in antigen presentation, T cell proliferation, and T cell survival [79]–[81]. Caution is therefore required when extrapolating these findings to natural infection with replicating parasites. Regardless, the finding that phagocytosis is insufficient to induce antigen presentation in this system highlights the importance of alternative approaches to deliver antigens for vaccine design and immunotherapies, such as those that target antigens to the host cell cytosol [82]. Furthermore, while many studies have utilized models of murine infection to elucidate the factors involved in the generation of T cell responses and the formation of memory T cells, vaccination with *cpsII* parasites allows these processes to be studied in a setting in which overt inflammation is limited. Thus, this experimental system may prove valuable to dissect basic principles that lead to the generation of long-lived T cell responses that translate easily to vaccine design, where inflammation should also be limited.

## Materials and Methods

### Ethics statement

All procedures involving mice were reviewed and approved by the Institutional Animal Care and Use Committee of the University of Pennsylvania (Animal Welfare Assurance Reference Number #A3079-01) and were in accordance with the guidelines set forth in the Guide for the Care and Use of Laboratory Animals of the National Institute of Health.

### Mice

Flt3L^−/−^ mice were obtained from Taconic Farms (Germantown, NY). Batf3^−/−^ mice, CCR2^−/−^ and CD11c-DTR mice were obtained from Jackson Laboratories. C57BL/6 mice were obtained from Jackson Laboratories or Taconic Farms. All mice were kept in specific-pathogen-free conditions at the School of Veterinary Medicine at the University of Pennsylvania. For experiments in which dendritic cells were depleted, CD11c-DTR or WT control mice were administered 100 ng of Diphtheria Toxin (Sigma-Aldrich) diluted in 100 µL of PBS (Invitrogen) intraperitoneally ∼12 hours prior to vaccination. Depletion efficiency was typically 90%.

### Infections

All experiments were performed using *cpsII* parasites, *cpsII*-OVA parasites [38], *cpsII*-OVA-mCherry parasites, or RH-OVA-Tomato parasites. RH-OVA-Tomato parasites [83] and *cpsII*-OVA parasites [38], [84] have been previously described. *CpsII*-OVA parasites and were derived from the RHΔ*cpsII* clone, which was provided as a generous gift by Dr. David Bzik [31]. *CpsII*-OVA-mCherry parasites were derived from the *cpsII*-OVA clone using the previously described methods [76], [77], with the exception that parasites were selected using zeomycin as previously described [85]. Parasites were cultured and maintained by serial passage on human foreskin fibroblast cells in the presence of parasite culture media [71.7% (Corning), 17.9% Medium 199 (Invitrogen), 9.9% Fetal Bovine Serum (FBS)(Invitrogen), 0.45% Penicillin and Streptomycin (Invitrogen)(final concentration of 0.05 units/ml Penicillin and 50 µg/ml Streptomycin), 0.04% Gentamycin (Invitrogen)(final concentration of 0.02 mg/ml Gentamycin)], which was supplemented with uracil (Sigma-Aldrich)(final concentration of 0.2 mM uracil) in the case of *cpsII*, *cpsII*-OVA and *cpsII*-OVA-mCherry parasites. For infections, parasites were harvested and serially passaged through 18, 20 and 26 gauge needles (BD) before filtration with a 5 µM filter (Sartorius Stedim). Parasites were washed extensively with PBS and mice were injected intraperitoneally with 10^5^ or 10^6^ parasites suspended in PBS. In vitro experiments were performed at an MOI of 0.5 or 1. For experiments in which CellTrace Violet (Invitrogen) was utilized to track the fate of parasites, CellTrace Violet was diluted in 200 µL of DMSO to obtain a 0.5 mM stock solution. Parasites were washed once with PBS before incubation in 0.5 µM CellTrace Violet diluted in PBS for 10–25 minutes at 37°C. This reaction was quenched by the addition of ∼40 volumes of complete media [88.5% RPMI 1640 (Corning), 8.8% FBS (Invitrogen), 0.9% Sodium Pyruvate (Gibco), 0.9% Penicillin and Streptomycin (Invitrogen)(final concentration of 0.1 units/ml Penicillin and 100 µg/ml Streptomycin), 0.9% MEM Non-essential Amino Acids Solution (Gibco) and 0.18% beta-2-mercaptoethanol (Gibco)] and parasites were washed extensively. In experiments in which 4-p-bromophenacyl bromide (4-p-bpb) was utilized to inhibit parasite invasion, 4-p-bpb (Sigma-Aldrich) was prepared fresh for each experiment and dissolved in DMSO (Sigma-Aldrich) to make a 0.1 M stock solution. Parasites were incubated in a 100 µM solution of 4-p-bpb in Fetal Bovine Serum at a concentration of 10^7^ parasites/ml for 10 minutes, and the reaction was quenched by the addition of ∼40 volumes of complete media, followed by extensive washing [12]. To heat-kill parasites, parasites were incubated at 60°C for 1 hour in PBS [86]. Death was confirmed using Trypan Blue staining (Corning).

### Cell culture and tissue harvesting

Peritoneal exudate cells were obtained by peritoneal lavage with 5 ml of PBS. Splenocytes and lymphocytes were obtained by grinding spleens and lymph nodes over a 40 µM filter (Biologix) and washing them in complete media. Red blood cells were then lysed by incubating for 5 minutes at room temperature in 5 ml of lysis buffer [0.864% ammonium chloride (Sigma-Aldrich) diluted in sterile de-ionized H_2_O)], followed by washing with complete media. Bone marrow-derived macrophages were obtained using previously described methods [83], [87]. Immortalized macrophages from C57BL/6 mice were obtained by transforming bone marrow-derived macrophages with the J2 Virus and were cultured in macrophage media [88].

### Flow cytometry and imaging

Tetramer-specific CD4^+^ T cells were measured using the protocol previously described [46]. MHCII Tetramer was obtained as generous gifts from Drs. Marc Jenkins and Marion Pepper, and subsequently from the NIH Tetramer Core Facility, and was used at a final concentration of 10 nM. APC-MHCI-SIINFEKL Tetramer was obtained from Beckman-Coulter. Cells were washed with FACS Buffer [1× PBS, 0.2% bovine serum antigen (Sigma), 1 mM EDTA (Invitrogen)], stained with LIVE/DEAD Fixable Aqua Dead Cell marker (Invitrogen) and incubated in Fc block [99.5% FACS Buffer, 0.5% normal rat serum (Invitrogen), 1 µg/ml 2.4G2 (BD)] prior to staining. The following antibodies were used for staining: Ki67 Alexa Fluor 488 (BD, B56), CD3 APC-eFluor 780 (eBioscience, 17A2), CD8 eFluor 450 (eBioscience, 53-6.7), CD11a PerCP-Cy5.5 (Biolegend, H155-78), MHCII PE (eBioscience, M5/114.15.2), NK1.1 PE (BD, PK136), CD19 PE (eBioscience, 1D3), Foxp3 eFlour 450 (eBioscience, FJK-16a), CD4 Pe-Cy7 (eBioscience, GK1.5), CD3 FITC (BD, 145-2C11), NK1.1 FITC (eBioscience, PK136), CD19 FITC (eBioscience, 1D3), Gr-1 PerCP-Cy5.5 (eBioscience, RB6-8C5), CD11c PE-Cy7 (eBioscience, N418), CD11b APC-eFluor 780 (eBioscience, M1/70), MHCII AF700 (Biolegend, M5/114.15.2), MHCI APC (AlexaFlour647 AF6-88.5), CD86 APC (eBioscience, GL1), CD40 APC (eBioscience 1C10), CD8 eFlour 650 NC (eBioscience, 53-6.7), CD45.2 APC-eFluor 780 (eBioscience, 104), polyclonal rabbit anti-*T. gondii* [a generous gift from Fausto G. Araujo (Palo Alto Medical Foundation, Palo Alto, CA)], and polyclonal Goat anti-Rabbit Alexa Fluor 680 (Jackson). Intracellular staining was performed using the Foxp3/Transcription Factor Staining Buffer Set (eBioscience) following the manufacturer's instructions. Samples were run on a FACSCanto (BD) or LSR Fortessa (BD) and analyzed using FlowJo Software (TreeStar). Images were obtained using the ImageStream and analysis was performed using IDEAS software (Amnis).

### Sorting

Splenic dendritic cells were obtained from mice injected subcutaneously with Flt3L-secreting b16 tumor cells [89], [90] and magnetically enriched using CD11c microbeads (Miltenyi Biotech) and LD MACS separation columns (Miltenyi Biotech), following the manufacturer's instructions. Bone marrow-derived dendritic cells were obtained by culturing bone marrow cells in the presence of 40 ng/ml of GM-CSF, which was added at days 0,3,6 and 9 post-seeding. Dendritic cells or bone marrow-derived macrophages were cultured overnight with parasites at 37°C and collected the following day. Dendritic cells were then stained for MHCII, CD11c, CD45, and free parasites, and sorted for mCherry^+ve^Violet^+ve^, mCherry^+ve^Violet^−ve^ or mCherry^−ve^Violet^−ve^ populations that were CD45^+^MHCII^HI^CD11c^HI^, and negative for free parasites using the FACSAria (BD). Macrophages were stained for CD45 and free parasites and sorted into mCherry^+ve^Violet^+ve^, mCherry^+ve^Violet^−ve^ or mCherry^−ve^Violet^−ve^ populations that were CD45^+^ and negative for free parasites.

### Electron microscopy

Bone marrow-derived macrophages from C57BL/6 mice were activated with IFN-γ and LPS for 18–24 hours or left untreated in macrophage media lacking uracil [DMEM (Gibco) supplemented with 4 mM L-glutamine (Sigma) and 10% dialyzed fetal bovine serum (Hyclone)]. Where indicated, cells were infected with freshly egressed parasites, washed three times with PBS then fixed at 2 hours or 24 hours post-infection. For ultrastructural analysis, cells were fixed in 2% paraformaldehyde/2.5% glutaraldehyde (Polysciences Inc., Warrington, PA) in 100 mM phosphate buffer, pH 7.2 for 1 hour at room temperature, processed and examined as described previously [91].

### Immunofluorescence assays

Immunofluorescence assays were performed in C57BL/6 bone marrow-derived macrophages. Bone marrow-derived macrophages for these experiments were derived as described previously [91]. Cells were activated with 100 U/ml IFN-γ and 0.1 ng/ml LPS in macrophage media lacking uracil. Macrophages were infected with freshly egressed parasites at an MOI of 1, washed at 3 hours post-infection five times with PBS, and incubated in uracil-free media supplemented with IFN-γ and LPS for the indicated time. Heat-killed parasites were incubated at 65°C for 10 minutes and infected at an MOI of 5. Cells for immunofluorescence were fixed in 4% formaldehyde, permeabilized with 0.05% saponin, and stained using primary antibodies as described. Parasite vacuoles were localized using mouse monoclonal Tg17-43 against GRA1 or rabbit polyclonal sera against GRA7. Host LAMP-1 was localized with rat monoclonal antibody 1D4B and Irgb6 was localized using rabbit polyclonal sera raised against recombinant protein [92]. All secondary antibodies used in immunofluorescence were highly-cross adsorbed Alexa Fluor conjugated antibodies (Invitrogen). Samples were visualized using a Zeiss Axioskop 2 MOT Plus microscope equipped for epifluorescence and using a 63× PlanApochromat lens, N.A. 1.40 (Carl Zeiss, Inc., Thornwood, NY). Images were acquired with an AxioCam MRm camera (Carl Zeiss, Inc.) using Axiovision v4.6, and processed using similar linear adjustments for all samples in Photoshop CS4 v9.

### Spinning disk confocal microscopy

Bone marrow-derived macrophages were cultured with invasion-blocked or untreated mCherry-expressing *cpsII* parasites (MOI = 1) and LysoTracker Green DND-26 (Life Technologies) was added prior to imaging, following the manufacturer's instructions. Images were collected using a Leica DMI4000 microscope equipped with a Yokogawa CSU10 spinning disk confocal unit and a Hamamatsu ImagEM EMCCD camera. Images were analyzed using ImageJ software.

### Statistical analysis

Statistical analysis was performed using PRISM software (Graphpad Software). Significance was calculated using an unpaired two-tailed student's t-test except when otherwise noted.

## Supporting Information

Figure S1 Images of mCherry^+ve^Violet^+ve^ (a) or mCherry^+ve^Violet^−ve^ (b–c) bone marrow-derived macrophages 18 hours following exposure to Violet-labeled, mCherry-expressing *cpsII* parasites, which were pre-treated with DMSO (a,b) or 4-p-bpb (c).
(PDF)

Figure S2 Subcellular localization of *cpsII* parasites. Invasion-blocked (4-p-bpb treated) or untreated mCherry-expressing *cpsII* parasites were incubated with bone marrow-derived macrophages for 1 hour or 9 hours, and acidified compartments were identified by staining with LysoTracker. Images were obtained by confocal microscopy.
(TIFF)

Figure S3 Images of mCherry^+ve^Violet^+ve^ (a) and mCherry^+ve^Violet^−ve^ (b,c) cells isolated from the PECS of mice 18 hours post-administration of 10^6^ DMSO-treated (a,b) or 4-p-bpb-treated (c) Violet-labeled, mCherry-expressing *cpsII* parasites.
(PDF)

Figure S4 CD4^+^ and CD8^+^ T cell responses to *cpsII*-OVA vaccination in Flt3L^−/−^ mice. Flt3L^−/−^ mice were vaccinated with 10^5^ *cpsII*-OVA parasites intraperitoneally and CD4^+^ and CD8^+^ T cell responses from the spleen and lymph nodes (pooled) were examined at 10 days post-vaccination. CD4^+^ T cell responses are shown (a). Flow plots shown in A are gated on Foxp3^−ve^ CD4^+^ T cells (top) or total CD4^+^ T cells (bottom), and the populations examined at the bottom of A were enriched for the tetramer^+ve^ population. CD8^+^ T cell responses in the spleen and lymph nodes (pooled) were also examined (b). Flow plots shown in B are gated on CD8^+^ T cells. *p<0.05; **p<0.005. ***p<0.0005. AVG±SE.
(EPS)

Figure S5 CD4^+^ and CD8^+^ T cell responses in WT and Batf3 KO mice. WT or Batf3 KO mice were vaccinated with 10^5^ *cpsII*-OVA parasites and examined 10 days post-vaccination. CD4^+^ T cell responses from the cells isolated from the spleen and lymph nodes (pooled) are shown (a). Flow plots in A are gated on Foxp3^−ve^CD4+ T cells (top), or total CD4^+^ T cells (bottom) and the populations examined in the bottom flow plots are enriched for the tetramer^+ve^ population. CD8^+^ T cell responses from cells isolated from the spleen and lymph nodes (pooled) are also shown (b). Flow plots in B are gated on CD8^+^ T cells. *P<0.05. CD4^+^ and CD8^+^ T cell data shown are from two separate experiments. AVG±SE.
(EPS)

Figure S6 CD4^+^ and CD8^+^ T cell responses in WT and CCR2^−/−^ mice. WT or CCR2^−/−^ mice were vaccinated with 10^5^ *cpsII*-OVA parasites and CD4^+^ and CD8^+^ T cell responses were examined 10 days post-infection. CD4^+^ T cell responses from cells isolated from the spleen are shown (a) and flow plots in A are gated on Foxp3^−ve^CD4+ T cells (top), or total CD4^+^ T cells (bottom) and the populations examined in the bottom flow plots are enriched for the tetramer^+ve^ population. CD8^+^ T cell responses from cells isolated from the spleen and lymph nodes (pooled) are also shown (b). Flow plots in B are gated on CD8^+^ T cells. *P<0.05. AVG±SE.
(EPS)

Figure S7 CD4^+^ and CD8^+^ T cell responses to live or heat-killed *cpsII*-OVA parasites. CD4^+^ and CD8^+^ T cell responses to 10^5^ untreated *cpsII*-OVA parasites, 10^5^ heat-killed *cpsII*-OVA parasites, or 10^7^ heat-killed *cpsII*-OVA parasites. Mice were administered parasites intraperitoneally, and CD4^+^ (a) and CD8^+^ (b) T cell responses were measured 10 days post-infection. Flow plots shown are gated on splenic CD4^+^ or CD8^+^ T cells. The populations depicted in the flow plots showing the tetramer-specific CD4^+^ T cells were enriched for the tetramer^+ve^ population. Parasite burden in the PECS is shown five days post-intraperitoneal challenge with 10^3^ tachyzoites of a highly virulent (RH) strain engineered to express OVA and the fluorescent protein dTomato, which was administered 3 weeks after vaccination with 10^5^ live *cpsII*-OVA parasites or 10^7^ heat-killed *cpsII*-OVA parasites (c).
(PDF)

Figure S8 Infected macrophages induce CD4^+^ and CD8^+^ T cell responses to *cpsII* parasites. Bone marrow-derived macrophages were harvested and incubated overnight with Violet-labeled, mCherry-expressing *cpsII* parasites and FACS-sorting was used the following day to isolate mCherry^+ve^Violet^+ve^ cells (infected cells) or mCherry^−ve^Violet^−ve^ (uninfected) cells. In parallel, 4-p-bpb-treated (invasion-blocked) parasites were incubated with bone marrow-derived macrophages and mCherry^+ve^Violet^−ve^ cells (cells that have phagocytosed parasites) were isolated by FACS sorting. 10^4^ cells from each of these populations were then administered to populations of mice and CD4^+^ (a) and CD8^+^ (b) T cell responses were measured 10 days post-transfer. Flow plots depicting total CD4^+^ T cell responses (a, top) are gated on CD3^+^CD4^+^Foxp3^−ve^ splenocytes and flow plots depicting tetramer-binding CD4^+^ T cells (a, bottom) are gated on CD3^+^CD4^+^ splenocytes. The population depicted in the flow plots demonstrating CD4^+^ tetramer binding is enriched for tetramer^+ve^ cells. Flow plots depicting CD8+ T cell responses (b) are gated on CD3^+^CD8^+^ splenocytes. Six weeks following the transfer of infected macrophages, uninfected macrophages, or macrophages that had phagocytosed *T. gondii*, mice were challenged with 10^3^ tachyzoites of a highly virulent, replicating strain of *T. gondii*, and parasite burden was measured in the PECS 5 days post-challenge (c).
(TIF)
