# Supplementary figures and images for: Parasite Fate and Involvement of Infected Cells in the Induction of CD4+ and CD8+ T Cell Responses to Toxoplasma gondii
Source: PLoS Pathog. 2014 Apr 10;10(4):e1004047. doi: 10.1371/journal.ppat.1004047 (PMC3983043; doi:10.1371/journal.ppat.1004047)

A mCherry+Violet+ parasites

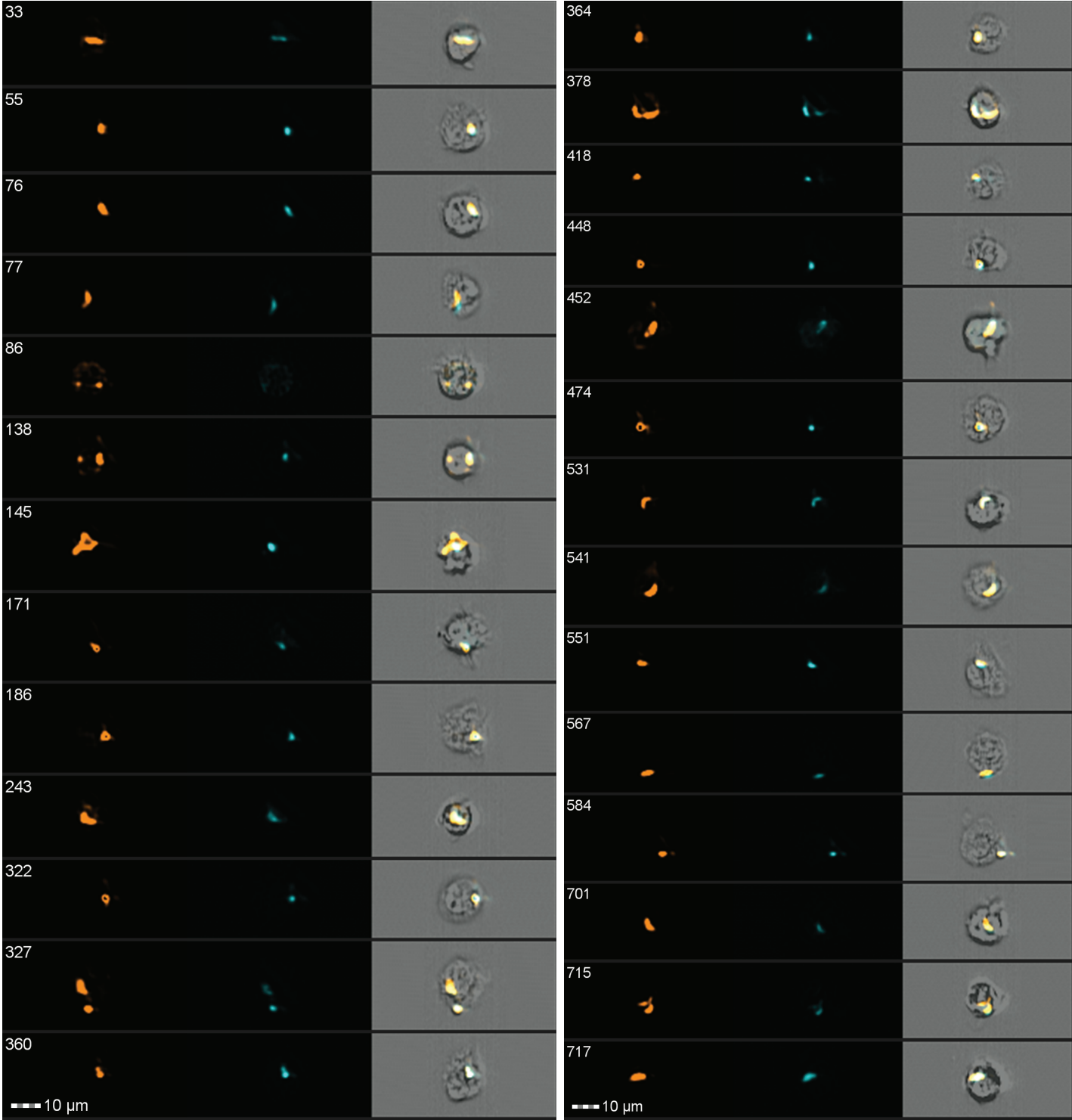

B mCherry+Violet- (Utx)

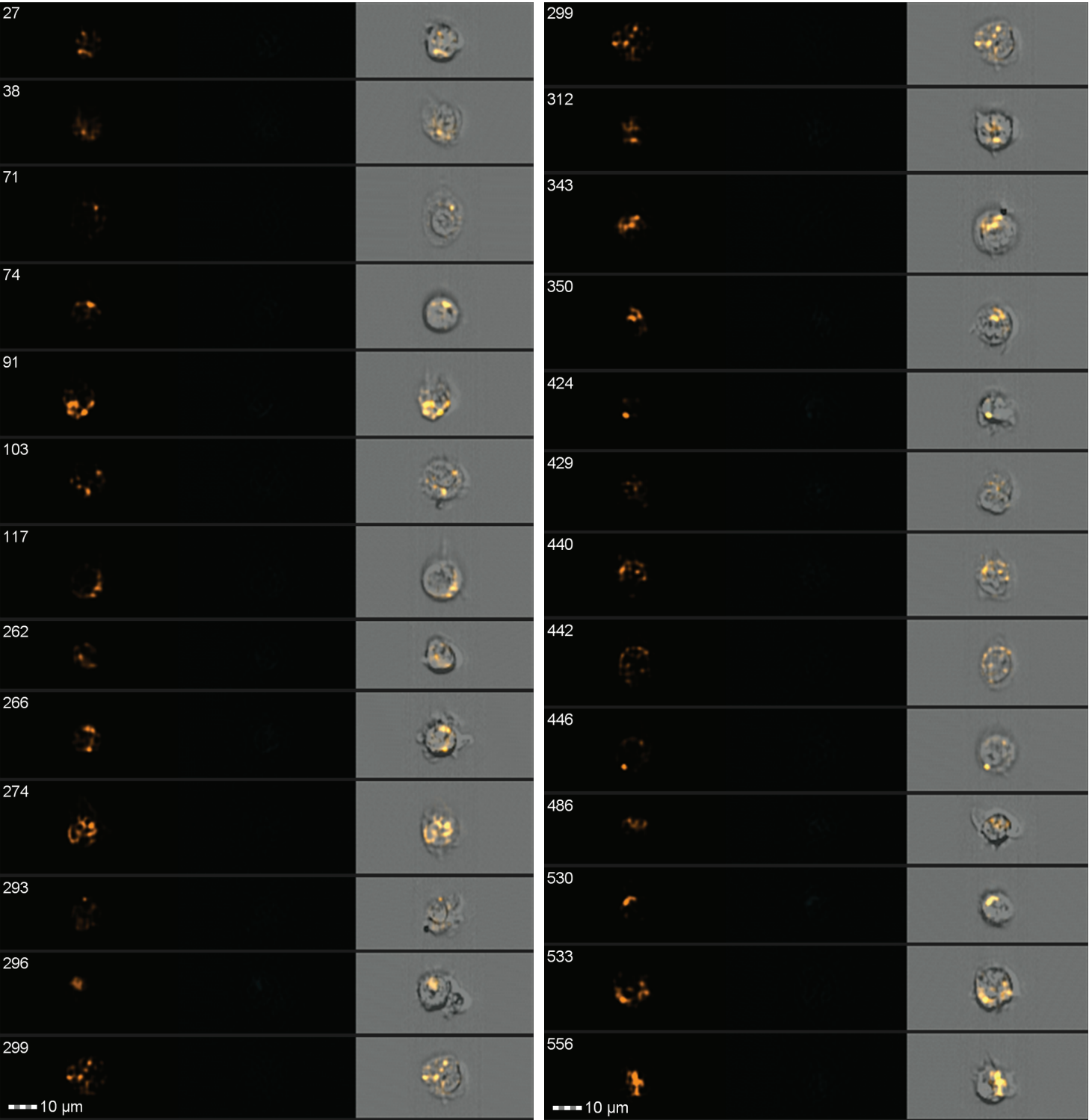

C mCherry+Violet- (4-p-bpb Tx)

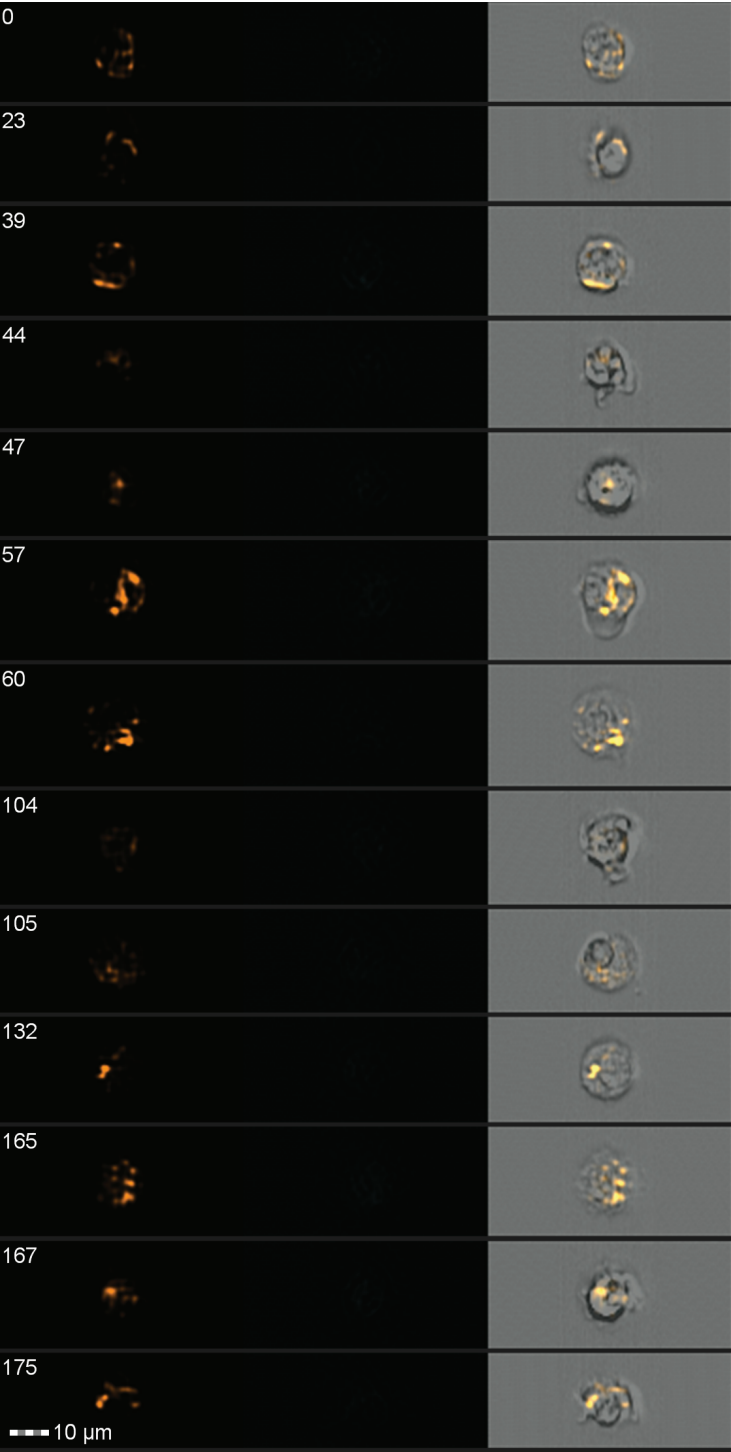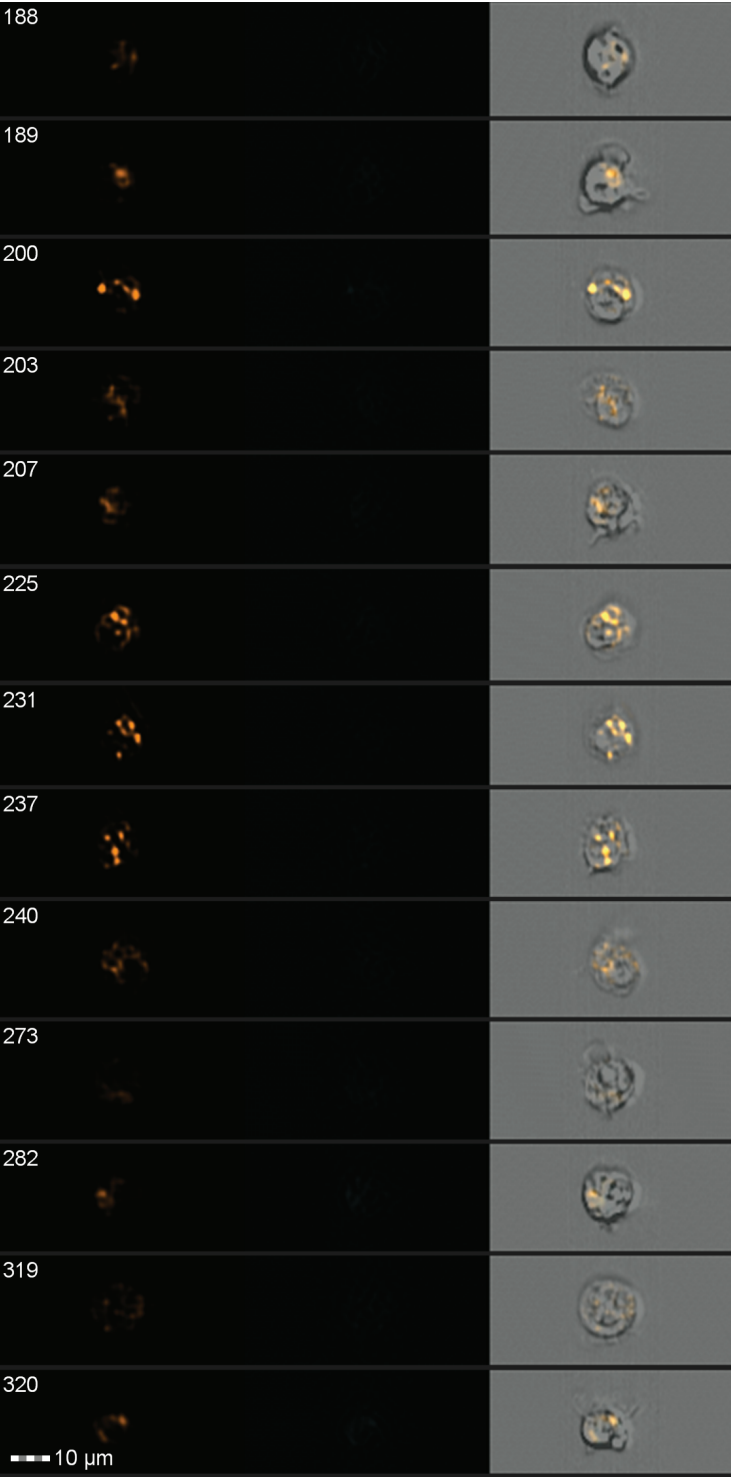

Supplement: Figure S1 — Images of mCherry+veViolet+ve (a) or mCherry+veViolet−ve (b–c) bone marrow-derived macrophages 18 hours following exposure to Violet-labeled, mCherry-expressing cpsII parasites, which were pre-treated with DMSO (a,b) or 4-p-bpb (c). (PDF) [file ppat.1004047.s001.pdf]

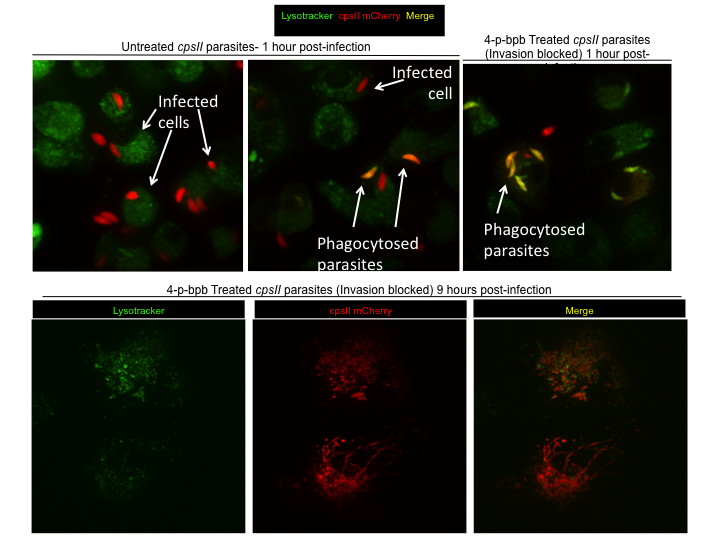

Supplement: Figure S2 — Subcellular localization of cpsII parasites. Invasion-blocked (4-p-bpb treated) or untreated mCherry-expressing cpsII parasites were incubated with bone marrow-derived macrophages for 1 hour or 9 hours, and acidified compartments were identified by staining with LysoTracker. Images were obtained by confocal microscopy. (TIFF) [file ppat.1004047.s002.tiff]

A mCherry+Violet+ (Utx)

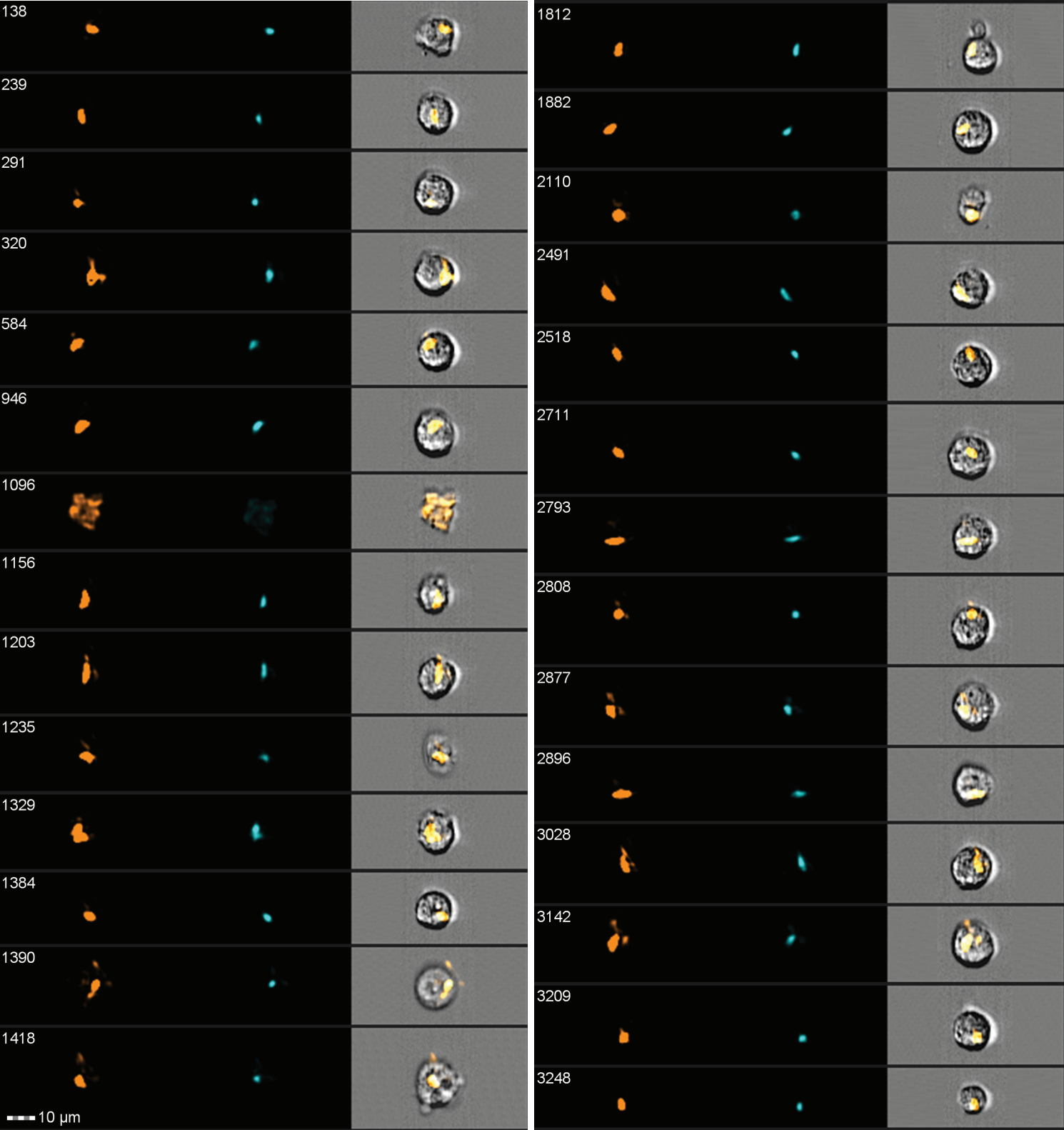

B mCherry+Violet- (Utx)

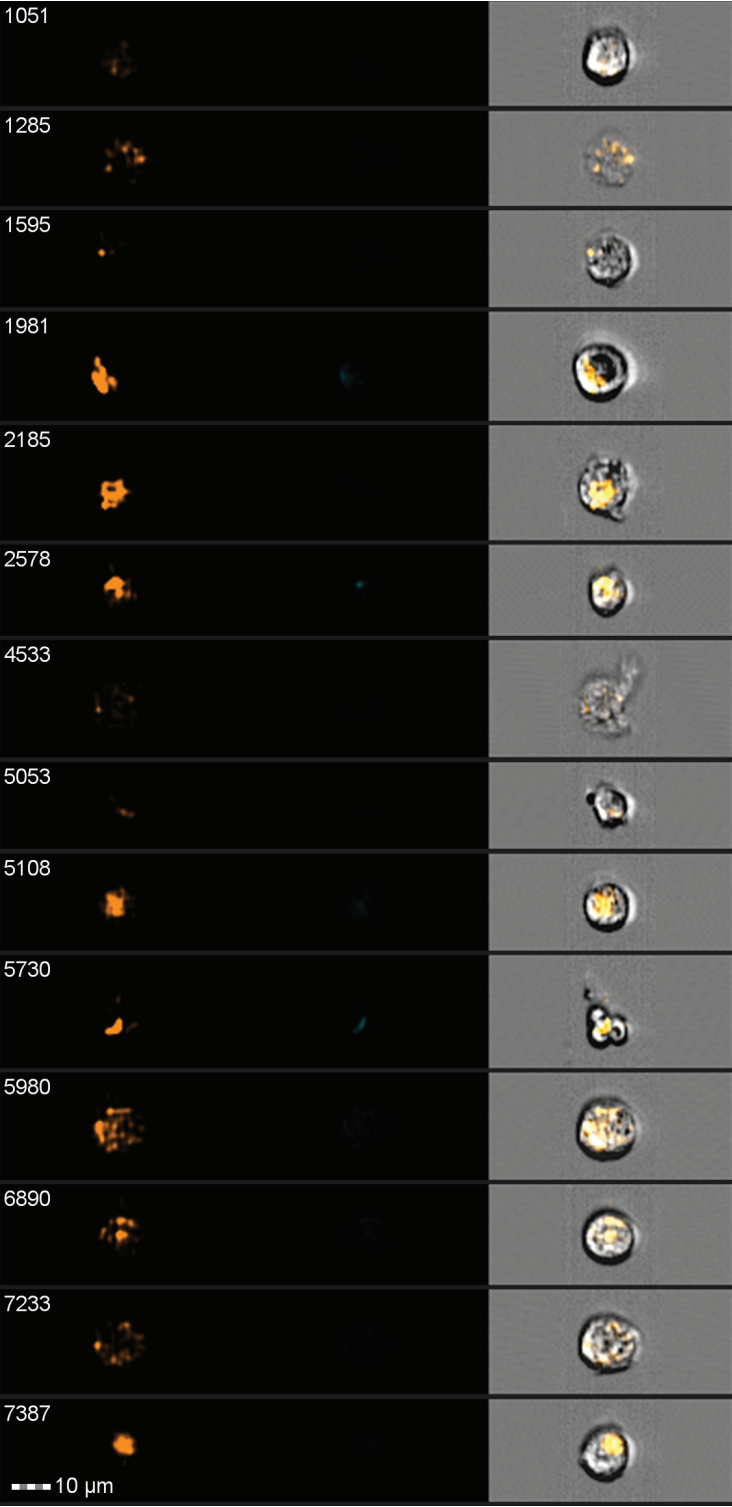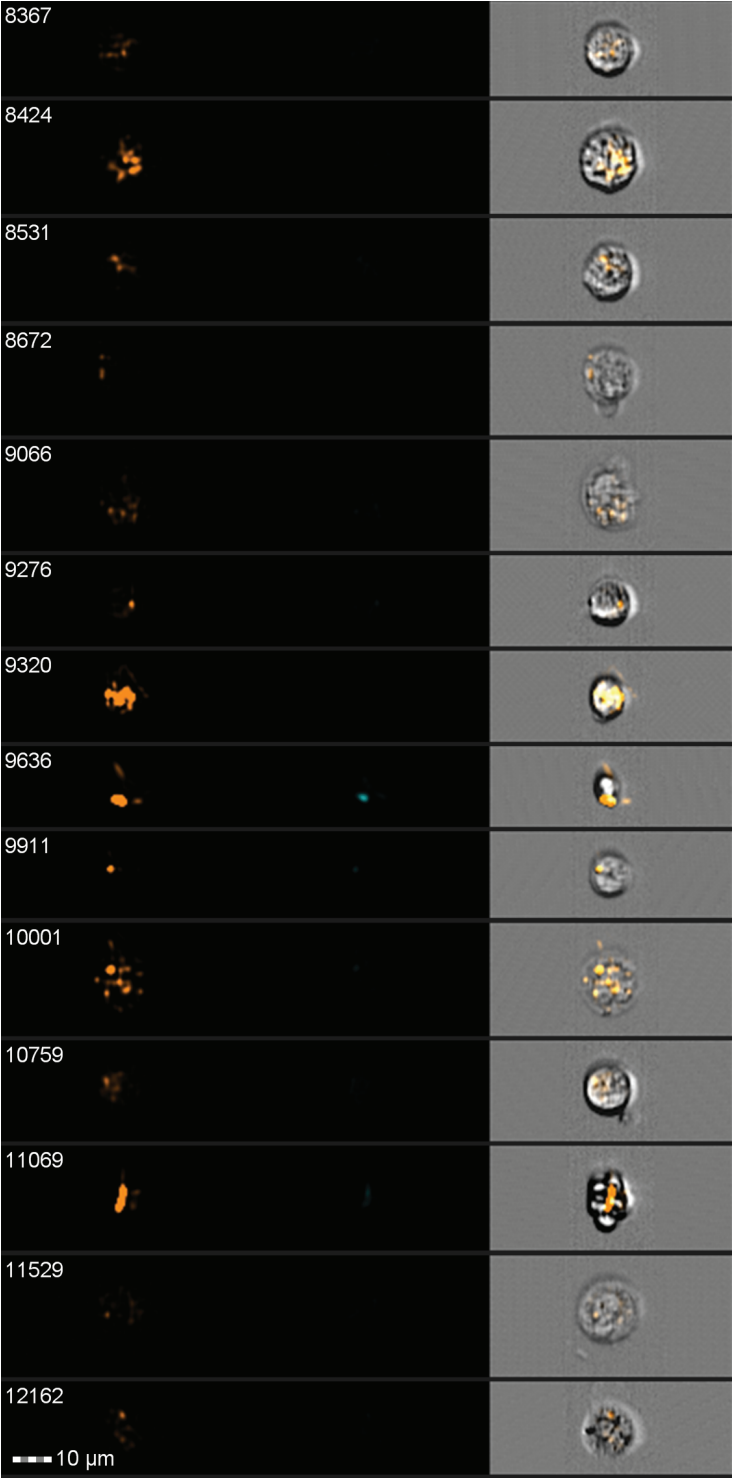

C mCherry+Violet- (4-p-bpb Tx)

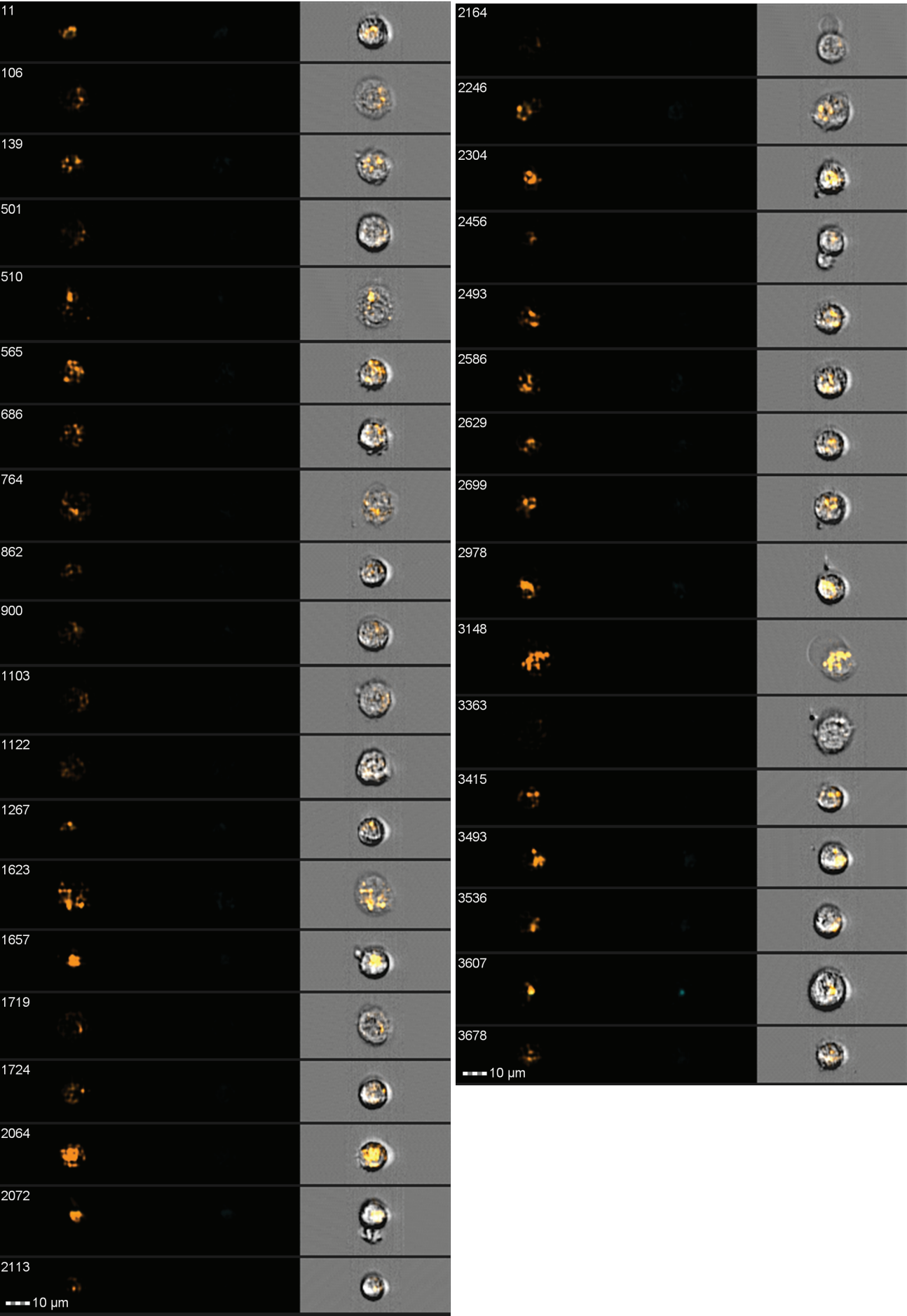

Supplement: Figure S3 — Images of mCherry+veViolet+ve (a) and mCherry+veViolet−ve (b,c) cells isolated from the PECS of mice 18 hours post-administration of 106 DMSO-treated (a,b) or 4-p-bpb-treated (c) Violet-labeled, mCherry-expressing cpsII parasites. (PDF) [file ppat.1004047.s003.pdf]

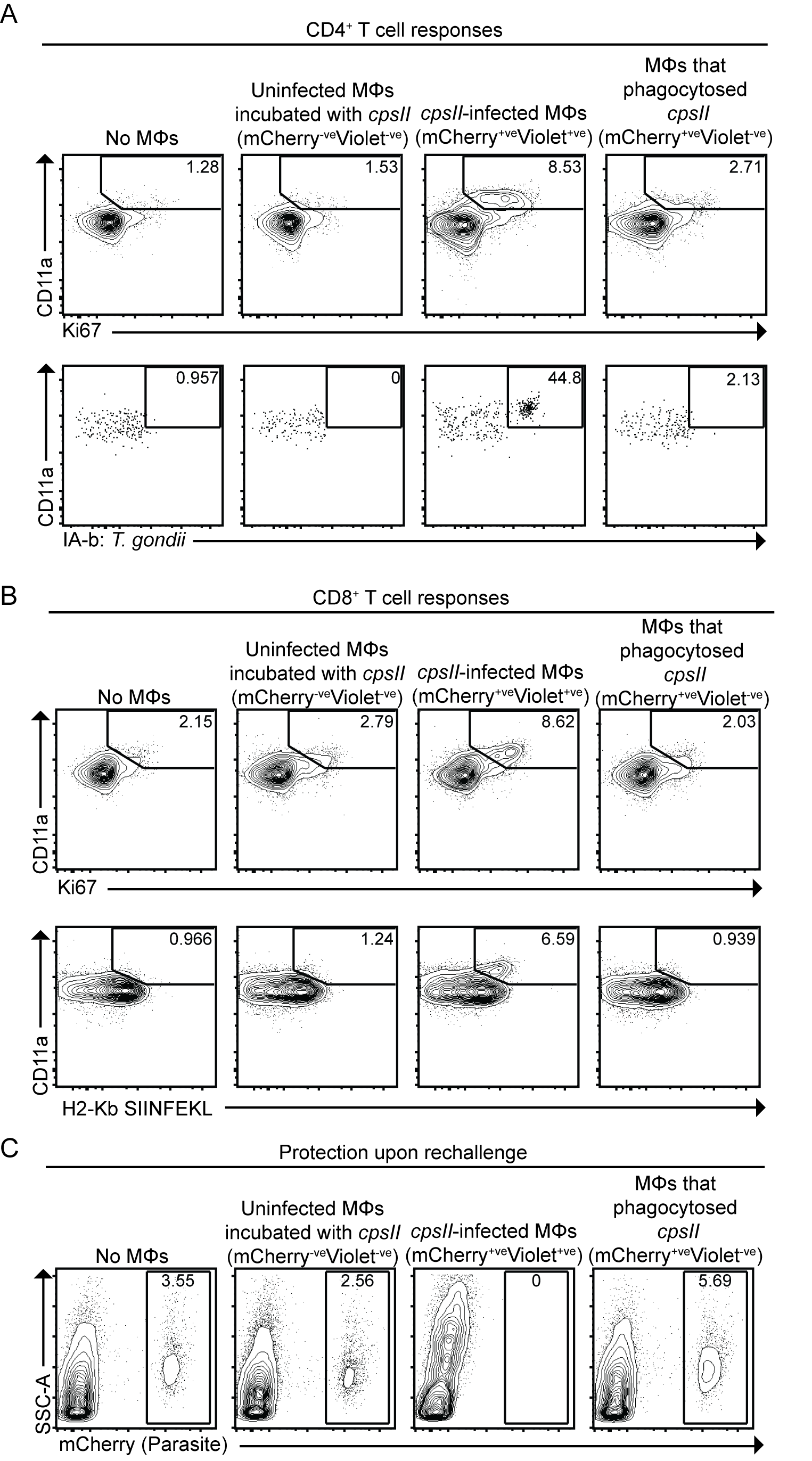

Supplement: Figure S8 — Infected macrophages induce CD4+ and CD8+ T cell responses to cpsII parasites. Bone marrow-derived macrophages were harvested and incubated overnight with Violet-labeled, mCherry-expressing cpsII parasites and FACS-sorting was used the following day to isolate mCherry+veViolet+ve cells (infected cells) or mCherry−veViolet−ve (uninfected) cells. In parallel, 4-p-bpb-treated (invasion-blocked) parasites were incubated with bone marrow-derived macrophages and mCherry+veViolet−ve cells (cells that have phagocytosed parasites) were isolated by FACS sorting. 104 cells from each of these populations were then administered to populations of mice and CD4+ (a) and CD8+ (b) T cell responses were measured 10 days post-transfer. Flow plots depicting total CD4+ T cell responses (a, top) are gated on CD3+CD4+Foxp3−ve splenocytes and flow plots depicting tetramer-binding CD4+ T cells (a, bottom) are gated on CD3+CD4+ splenocytes. The population depicted in the flow plots demonstrating CD4+ tetramer binding is enriched for tetramer+ve cells. Flow plots depicting CD8+ T cell responses (b) are gated on CD3+CD8+ splenocytes. Six weeks following the transfer of infected macrophages, uninfected macrophages, or macrophages that had phagocytosed T. gondii, mice were challenged with 103 tachyzoites of a highly virulent, replicating strain of T. gondii, and parasite burden was measured in the PECS 5 days post-challenge (c). (TIF) [file ppat.1004047.s008.tif]
