# Supplementary material for: Parasite Fate and Involvement of Infected Cells in the Induction of CD4+ and CD8+ T Cell Responses to Toxoplasma gondii
Source: PLoS Pathog. 2014 Apr 10;10(4):e1004047. doi: 10.1371/journal.ppat.1004047 (PMC3983043; doi:10.1371/journal.ppat.1004047)
Supplement: Figure S7 — CD4+ and CD8+ T cell responses to live or heat-killed cpsII-OVA parasites. CD4+ and CD8+ T cell responses to 105 untreated cpsII-OVA parasites, 105 heat-killed cpsII-OVA parasites, or 107 heat-killed cpsII-OVA parasites. Mice were administered parasites intraperitoneally, and CD4+ (a) and CD8+ (b) T cell responses were measured 10 days post-infection. Flow plots shown are gated on splenic CD4+ or CD8+ T cells. The populations depicted in the flow plots showing the tetramer-specific CD4+ T cells were enriched for the tetramer+ve population. Parasite burden in the PECS is shown five days post-intraperitoneal challenge with 103 tachyzoites of a highly virulent (RH) strain engineered to express OVA and the fluorescent protein dTomato, which was administered 3 weeks after vaccination with 105 live cpsII-OVA parasites or 107 heat-killed cpsII-OVA parasites (c). (PDF) [file ppat.1004047.s007.pdf]

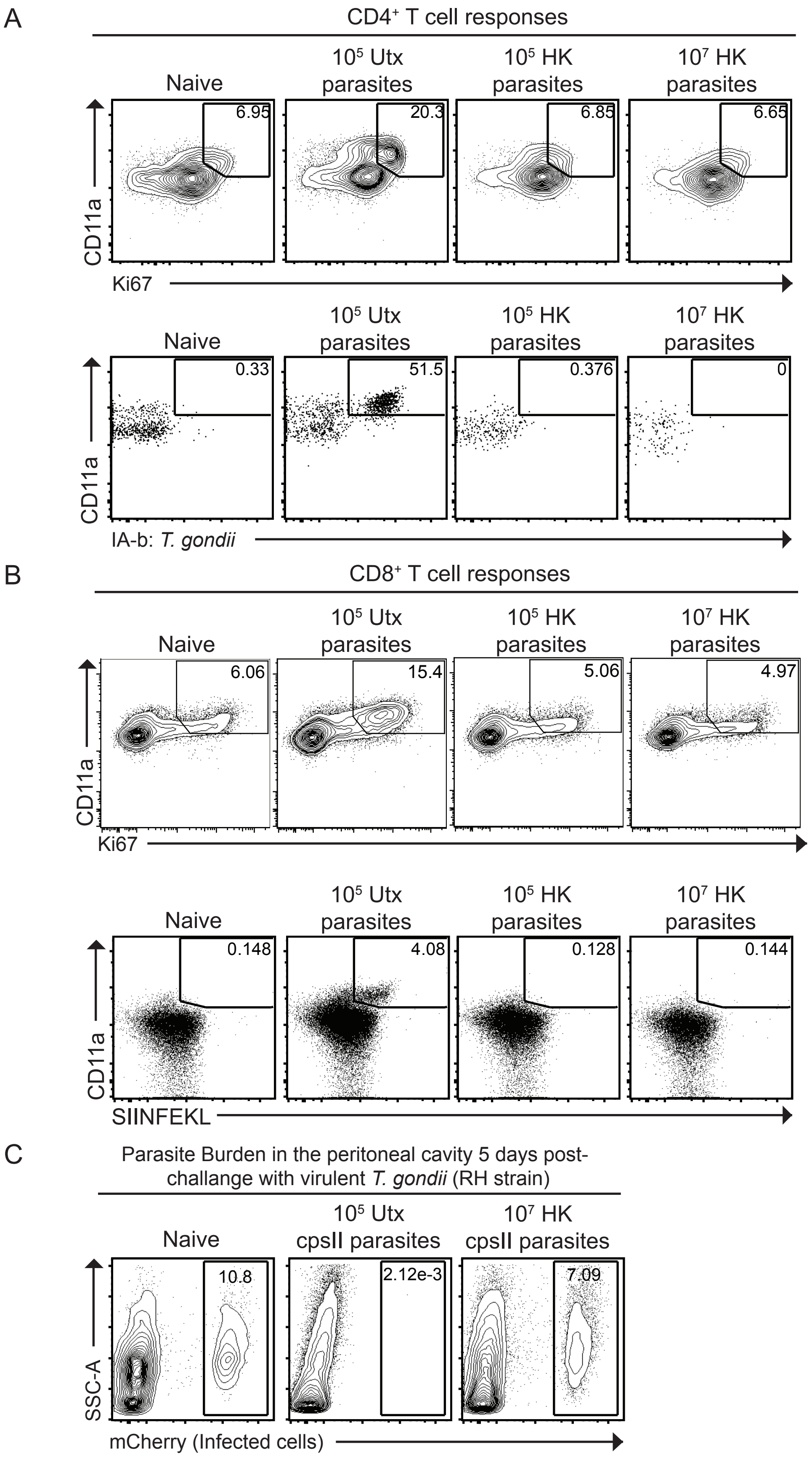
A
CD4+ T cell responses
Naive
10^5 Utx parasites
10^5 HK parasites
10^7 HK parasites
6.95
20.3
6.85
6.65
CD11a
Ki67
0.33
51.5
0.376
0
IA-b: T. gondii
B
CD8+ T cell responses
6.06
15.4
5.06
4.97
0.148
4.08
0.128
0.144
SIINFEKL
C
Parasite Burden in the peritoneal cavity 5 days post-challange with virulent T. gondii (RH strain)
10^5 Utx cpsII parasites
10^7 HK cpsII parasites
10.8
2.12e-3
7.09
SSC-A
mCherry (Infected cells)
